# fMiRNA-192 and miRNA-204 Directly Suppress lncRNA HOTTIP and Interrupt *GLS1*-Mediated Glutaminolysis in Hepatocellular Carcinoma

**DOI:** 10.1371/journal.pgen.1005726

**Published:** 2015-12-28

**Authors:** Yunxia Ge, Xiaodan Yan, Yiguang Jin, Xinyu Yang, Xiang Yu, Liqing Zhou, Sichong Han, Qipeng Yuan, Ming Yang

**Affiliations:** 1 State Key Laboratory of Chemical Resource Engineering, Beijing Laboratory of Biomedical Materials, College of Life Science and Technology, Beijing University of Chemical Technology, Beijing, China; 2 Department of Pharmaceutical Sciences, Beijing Institute of Radiation Medicine, Beijing, China; 3 Department of Radiation Oncology, Huaian No. 2 Hospital, Huaian, Jiangsu Province, China; University of Basel, Università del Molise, SWITZERLAND

## Abstract

Accumulated evidence demonstrated that long non-coding RNAs (lncRNAs) play a pivotal role in tumorigenesis. However, it is still largely unknown how these lncRNAs were regulated by small ncRNAs, such as microRNAs (miRNAs), at the post-transcriptional level. We here use lncRNA HOTTIP as an example to study how miRNAs impact lncRNAs expression and its biological significance in hepatocellular carcinoma (HCC). LncRNA HOTTIP is a vital oncogene in HCC, one of the deadliest cancers worldwide. In the current study, we identified miR-192 and miR-204 as two microRNAs (miRNAs) suppressing HOTTIP expression via the Argonaute 2 (AGO2)-mediated RNA interference (RNAi) pathway in HCC. Interaction between miR-192 or miR-204 and HOTTIP were further confirmed using dual luciferase reporter gene assays. Consistent with this notion, a significant negative correlation between these miRNAs and HOTTIP exists in HCC tissue specimens. Interestingly, the dysregulation of the three ncRNAs was associated with overall survival of HCC patients. In addition, the posttranscriptional silencing of HOTTIP by miR-192, miR-204 or HOTTIP siRNAs could significantly suppress viability of HCC cells. On the contrary, antagonizing endogenous miR-192 or miR-204 led to increased HOTTIP expression and stimulated cell proliferation. *In vivo* mouse xenograft model also support the tumor suppressor role of both miRNAs. Besides the known targets (multiple 5’ end HOX A genes, i.e. *HOXA13*), *glutaminase* (*GLS1*) was identified as a potential downstream target of the miR-192/-204-HOTTIP axis in HCC. Considering glutaminolysis as a crucial hallmark of cancer cells and significantly inhibited cell viability after silencingGLS1, we speculate that the miR-192/-204-HOTTIP axis may interrupt HCC glutaminolysis through GLS1 inhibition. These results elucidate that the miR-192/-204-HOTTIP axis might be an important molecular pathway during hepatic cell tumorigenesis. Our data in clinical HCC samples highlight miR-192, miR-204 and HOTTIP with prognostic and potentially therapeutic implications.

## Author Summary

Accumulated evidence demonstrated that long non-coding RNAs (lncRNAs) play a pivotal role in tumorigenesis. Here, we for the first time demonstrated how microRNAs (miRNAs) impact onco-lncRNA HOTTIP expression and its biological significance in hepatocellular carcinoma (HCC). We identified miR-192 and miR-204 as two miRNAs suppressing HOTTIP expression via the Argonaute 2-mediated RNA interference pathway. The dysregulation of the three ncRNAs was associated with overall survival of HCC patients. The posttranscriptional silencing of HOTTIP by miR-192, miR-204 or HOTTIP siRNAs could significantly suppress viability of HCC cells *in vitro* and *in vivo*. Besides one of the known target gene *HOXA13*, *glutaminase* was identified as a potential downstream target of the miR-192/-204-HOTTIP axis in HCC. Our data will have high impact on our understanding of how miRNAs are involved in the fine-regulation of lncRNAs and the potential translation in clinic.

## Introduction

Hepatocellular carcinoma (HCC) ranks among the 10 most common cancers worldwide and showed the highest incidence in Asia [1,2]. Remarkably, more than half of all HCC patients were diagnosed in China [1]. Chronic infection with the hepatitis B or C viruses (HBV or HCV), exposure to dietary aflatoxin B as well as alcohol abuse have been identified as major risk factors of this malignancy. However, only a portion of exposed individuals finally developed HCC, indicating that genetic makeup may also contribute to HCC etiology [1,2].

Long noncoding RNAs (lncRNAs) constitute a class of endogenous RNAs ranging in size from several hundred to tens of thousands of nucleotides (nt) [3–5]. Different from their shorter counterparts, such as microRNAs (miRNAs), the role of most lncRNAs in human cancers is still largely unknown. Accumulating data have established the participation of several lncRNAs during tumorigenesis and progression of HCC. For instance, lncRNA HOTTIP, HULC, MALAT1, HOTAIR, lncRNA-HEIH, HBx-LINE1 and lncRNA-hPVT1show their capability to promote HCC proliferation as oncogenes [6–14]. Conversely, lncRNA H19, MEG3 and lncRNA-Drehmay act as tumor suppressors [15–17]. In addition, multiple lncRNAs (i.e. lncRNA-LET, lncRNA-ATB, lncRNA-Dreh, MALAT1, HOTAIR and MVIH) are involved in controlling HCC invasion and metastasis [10,11,17–20].

The HCC-related lncRNA HOTTIP is a 3764 nt, spliced and polyadenylated ncRNA, which is transcribed from 330 bases upstream of the 5’ tip of*HOXA13* (Chromosome 7p15.2) [6,21]. During development, HOTTIP RNA is mainly expressed in distal anatomic sites and controls activation of distal *HOXA* genes *in vivo* [21]. Through directly binding the adaptor protein WDR5of the WDR5/MLL complex, HOTTIP drives histone H3 lysine 4 trimethylation (H3K4me3) and gene transcription across the *HOXA* gene locus. In mice, HOTTIP knockout leads to defects of resembling HoxA11 andHoxA13 inactivation, demonstrating its essential part in controlling development of lumbo-sacral anatomic regions [21]. After analyzing 52 snap-frozen needle HCC biopsies and matched non-neoplastic counterparts, Quagliata et al found that HOTTIP is significantly up-regulated in HCC and HOTTIP/HOXA13 expression is associated with patients’ metastasis and survival. Additional gain and loss of function experiments demonstrated that silencing HOTTIP inhibits HCC proliferation, highlighting its role as an oncogene during hepatocarcinogenesis [6]. However, fine regulation of lncRNA HOTTIP expression in HCC is still largely unknown.

Intriguingly, miRNAs may directly interact with lncRNAs and knock-down their expression [22,23]. However, how HOTTIP is regulated by miRNAs at the posttranscriptional level remains largely unclear. In the current study, we for the first time identified the negative regulation of lncRNA HOTTIP by miR-192 and miR-204 via the Argonaute 2 (AGO2)-mediated RNAi pathway. Ectopic expression of miR-192/-204 or HOTTIP siRNA significantly suppresses glutaminase (GLS1) expression, thereby inhibiting HCC growth *in vitro* and *in vivo*.

## Results

### Identification of candidate miRNAs targeting HOTTIP

**Fig 1:**
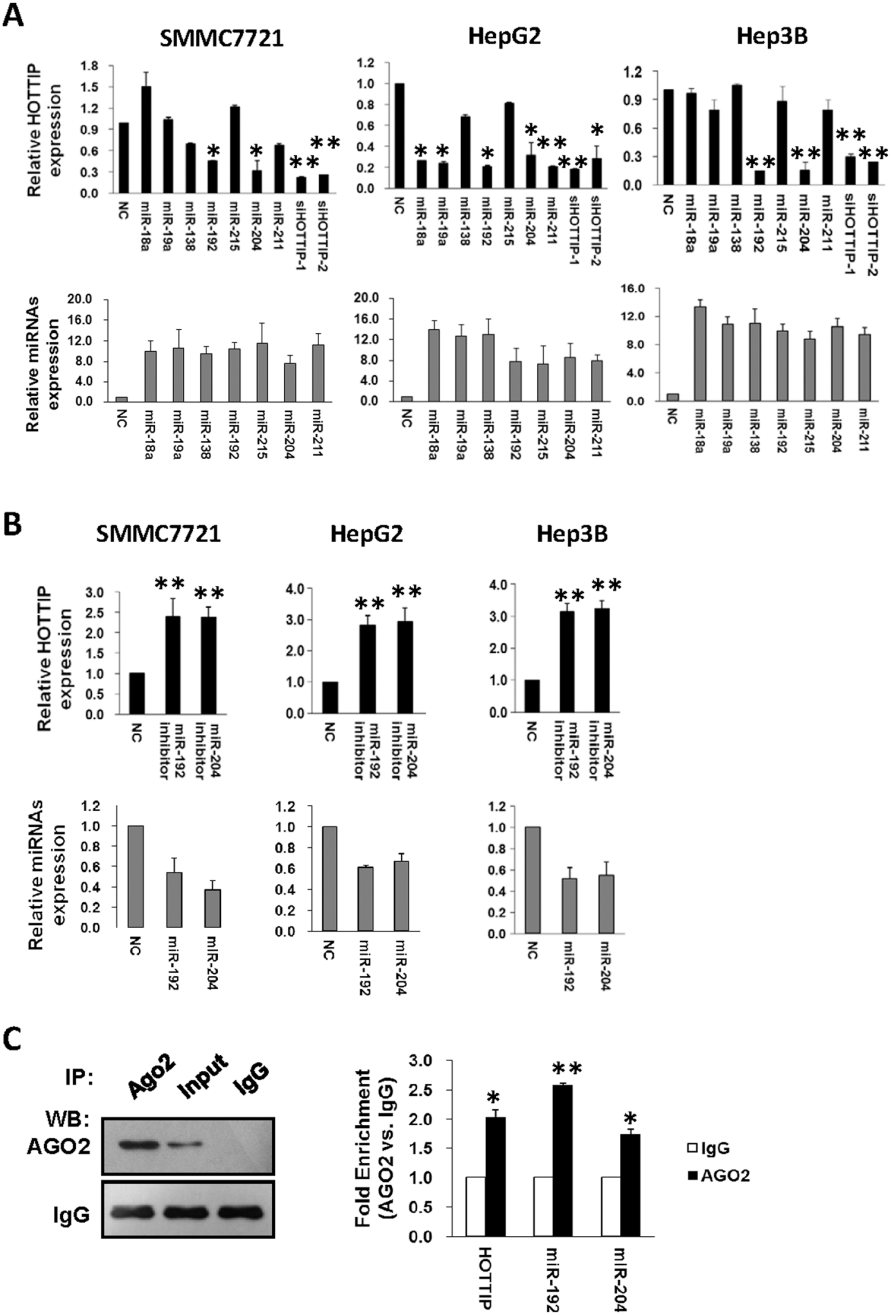
Identification of candidate miRNAs targeting lncRNA HOTTIP. (A) MiR-192 and miR-204 inhibit HOTTIP expression. 20 nmol/L mimics of miR-138, miR-18, miR-192, miR-215, miR-19, miR-204, and miR-211, two HOTTIP siRNAs (siHOTTIP-1 and siHOTTIP-2)or NC RNA were transfected into SMMC7721,HepG2 and Hep3B HCC cells. HOTTIP expression was detected at 48h after transfection (up, black columns). The transfection efficiency of different miRNAs was confirmed by qRT-PCR (low, grey columns). (B) Inhibition of miR-192 or miR-204 up-regulates HOTTIP expression. 20 nmol/L miR-192 inhibitors, miR-204 inhibitors and NC RNA were transfected into SMMC7721,HepG2 and Hep3BHCC cells. HOTTIP and miRNAs expression was detected at 48h after transfection (up, black columns). The transfection efficiency of different miRNAs was confirmed by qRT-PCR (low, grey columns). (C) Association of miR-192/-204 and HOTTIP with AGO2. HepG2 cellular lysates were used for RNA immunoprecipitation with AGO2 antibody. Detection of AGO2 and IgG using western blot (up), and detection of miR-192, miR-204 or HOTTIP using qRT-PCR (low). All data of HOTTIP expression were normalized to *β-actin* mRNA expression levels. All miRNA expression data were normalized to U6 small RNA expression. All results of the mean of triplicate assays with standard deviation are presented.*P< 0.05, **P< 0.01.

Potential miRNA candidates targeting HOTTIP was predicted by the miRCode software (www.miRCode.org)[24]. As shown in S1 Table, there are a total of 62 miRNA matching sites in *HOTTIP* gene. Considering that matching sites with higher evolutionary conservation across species might be more functionally vital, we evaluated seven candidate miRNAs (miR-138, miR-18, miR-192, miR-215, miR-19, miR-204, and miR-211) which show >80% conservation among primates (8 species excluding human) in this study (Table 1). After transfection with the RNA mimics of the seven miRNAs and two HOTTIP siRNAs (siHOTTIP-1 and siHOTTIP-2) into SMMC7721, HepG2 and Hep3B cells, we firstly examined endogenous lncRNA *HOTTIP* expression changes (Fig 1A). About 70~80% decreased HOTTIP expression was observed in HCC cells with overexpression of siHOTTIP-1 or siHOTTIP-2 compared to NC-RNA-transfected cells (both *P*<0.01). In HepG2 cells, miR-192, miR-204, miR-18, miR-19 and miR-211 could down-regulate HOTTIP expression (all *P*<0.05). In SMMC7721 cells, miR-192 and miR-204 can significantly inhibit HOTTIP expression (both *P*<0.05). Similar results were also observed in Hep3B cells. Considering the consistency in all three cell lines, we only investigated miR-192 and miR-204 in the following studies. To further validate this regulatory relationship, inhibitors of both miRNAs were delivered into SMMC7721,HepG2 and Hep3Bcell lines to antagonizeendogenousmiR-192/-204 expression. As expected, significant increased HOTTIP levels could be found in HCC cells (all *P*<0.05) (Fig 1B).

**Table 1:** Bioinformatics prediction of miRNAs regulating lncRNA HOTTIP at highly conserved RNA sequences.*

MicroRNAs	Seed position	Conservation		
		Primates	Mammals	Other Vertebrates
miR-138	chr7:27245289	89%	30%	0%
miR-18	chr7:27238346	89%	30%	0%
miR-192/215	chr7:27241747	89%	91%	77%
miR-19	chr7:27245115	100%	0%	0%
miR-204/211	chr7:27245995	89%	43%	0%

*Data from miRcode (http://www.mircode.org/mircode/).

Since miR-192 and miR-204 showed similar regulatory behaviors as HOTTIP siRNAs, we speculated that miR-192/-204 may regulate HOTTIP expression via the RNAi pathway at the post-transcriptional level. This would suggest that both miR-192/-204 and HOTTIP exist in the RISC complex(RNA-induced silencing complex). Since AGO2 is a key component of the RISC complex, we therefore performed RNA immunoprecipitation with the AGO2 antibody. We firstly confirmed that the AGO2 protein could be precipitated from the cellular extract(Fig 1C). In RNA extracted from the precipitated AGO2 protein, we could detect both miR-192/-204 and HOTTIP with a2~2.5-folds enrichment compared to IgG (Fig 1C).

### Interaction of miR-192/-204 and lncRNA HOTTIP

**Fig 2:**
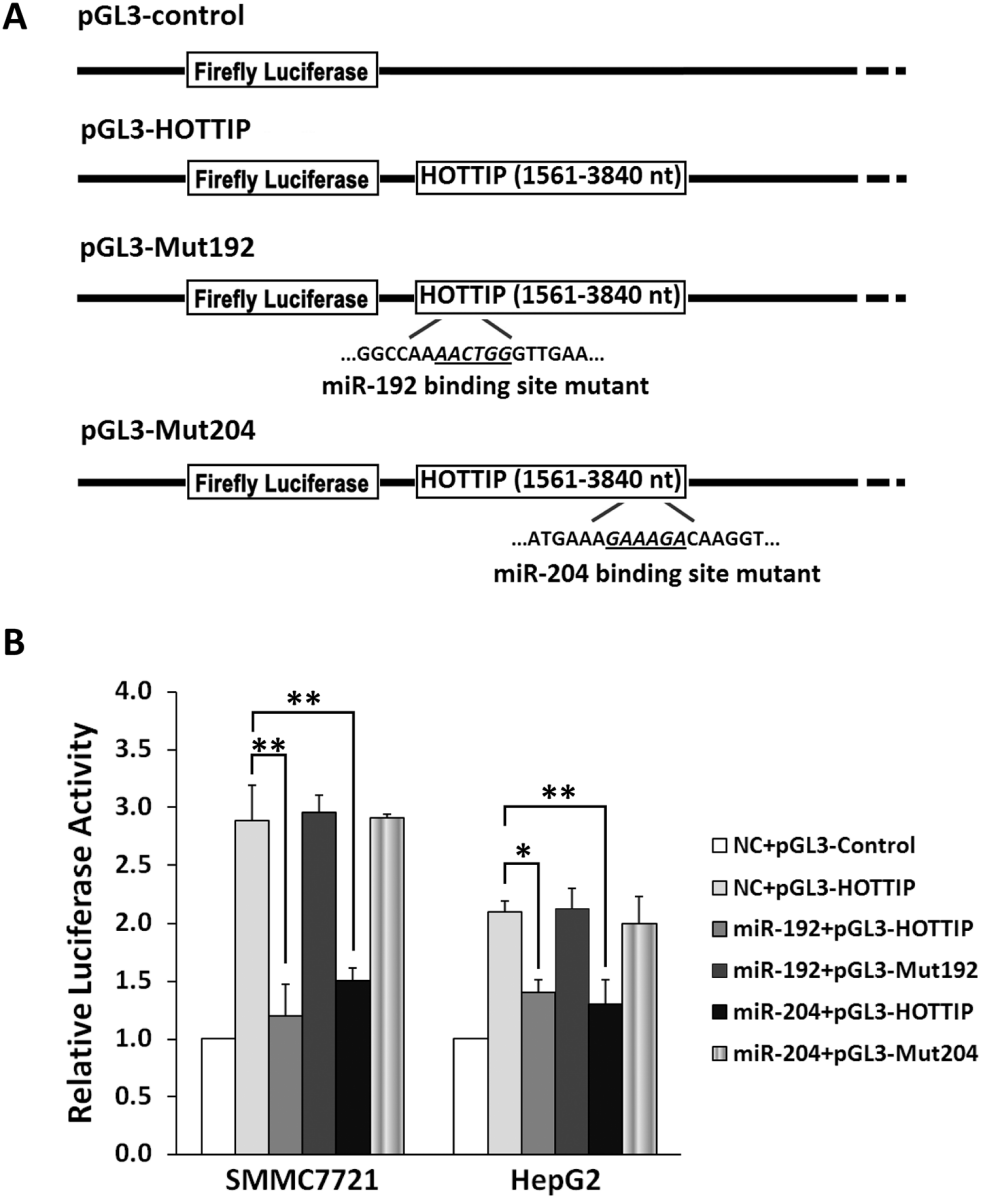
MiR-192 and miR-204 regulate HOTTIP expression in a posttranscriptional manner. (A) Schematic constructions of pGL3-HOTTIP (HOTTIP 3' partial region), pGL3-Mut192 and pGL3-Mut204. (B) pGL3-HOTTIP, pGL3-Mut192 or pGL3-Mut204 was co-transfected into SMMC7721 and HepG2 cells withmiR-192 mimics, miR-204 mimics or NC RNA. Luciferase activity was detected at 48h after transfection and normalized relative to the Renilla luciferase expression. Inhibition effects of miR-192 mimics or miR-204 mimics on pGL3-HOTTIP, pGL3-Mut192 or pGL3-Mut204 were showed. All results of the mean of triplicate assays with standard deviation of the mean are presented. *P< 0.05, **P< 0.01.

To examine the potential miRNA-lncRNA interaction, a 2280bp human *HOTTIP*3' partial region was subcloned after the firefly luciferase gene (named as pGL3-HOTTIP) (Fig 2A)and co-transfected into SMMC7721 and HepG2 cells withmiR-192 mimics,miR-204 mimics or NC RNA. MiR-192 produced a 58.3% or33.3% decreased luciferase activity compared to NC RNAinSMMC7721 or HepG2cells (both *P*<0.05)(Fig 2B). Similarly, there was a 47.9%or 38.1% decrease in luciferase activity in both cell lines with miR-204overexpression compared to NC RNA (both *P*<0.01)(Fig 2B). An analogous reporter with point substitutions disrupting the target sites of miR-192 or miR-204(named as pGL3-Mut192 or pGL3-Mut204) (Fig 2A) was also co-transfected with two miRNA mimics or NC RNA. However, no significant decreased luciferase activity caused by miR-192 or miR-204was observed compared to NC RNA(all *P*>0.05)(Fig 2B).

### MiR-192 and miR-204 inhibit HCC proliferation via suppressing lncRNA HOTTIP

**Fig 3:**
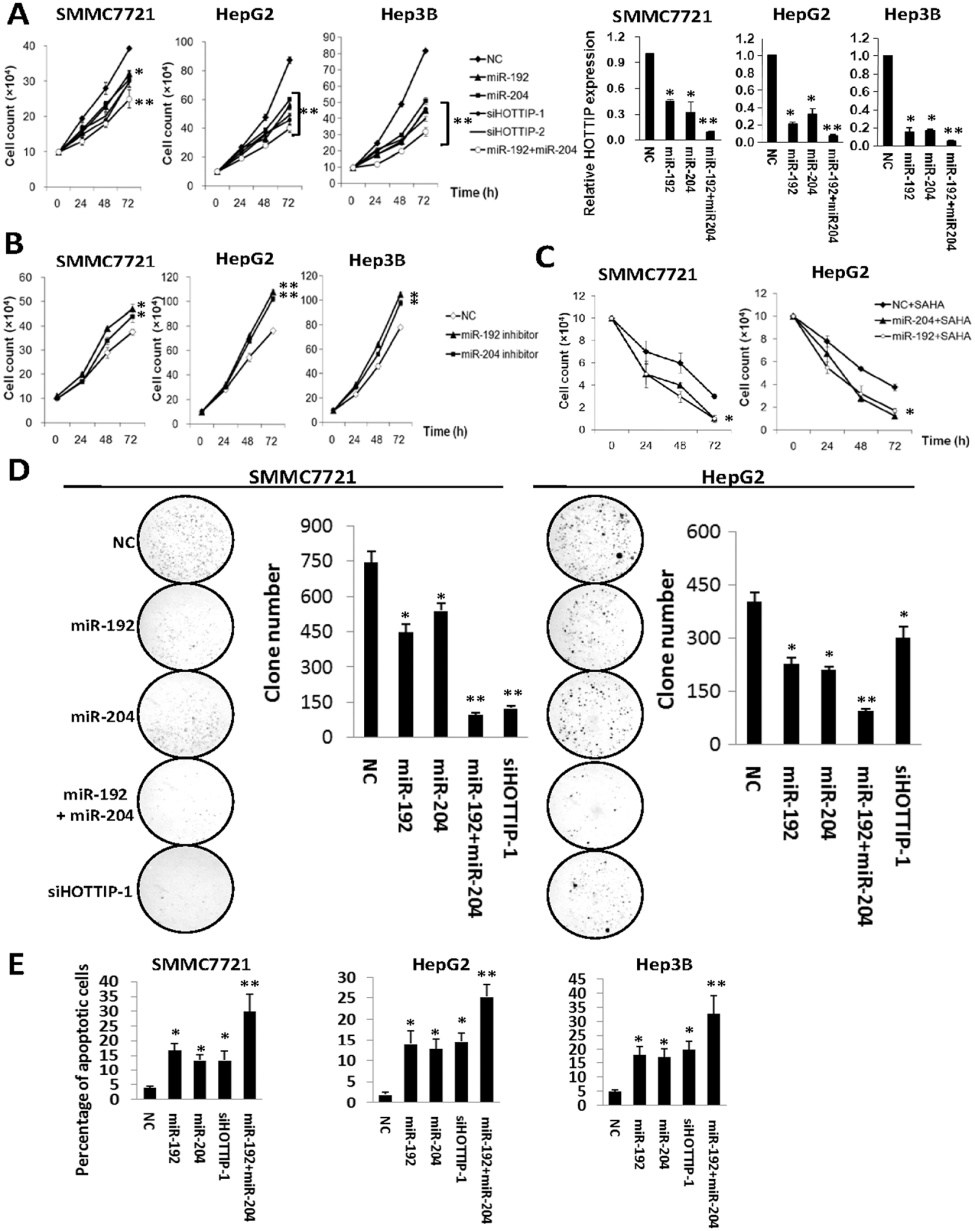
MiR-192 and miR-204 additively inhibits cell proliferation ofSMMC7721, HepG2 orHep3B cells. 20 nmol/L miR-192, miR-204, NC RNA or HOTTIPsiRNAs was transfected into HCC cells, respectively. 10 nmol/L miR-192 mimics and 10 nmol/L miR-204 mimics were co-transfected into HCC cells. (A) Overexpression of miR-192, miR-204 or two HOTTIP siRNAs (siHOTTIP-1 and siHOTTIP-2) inhibits HCC cell growth. Cell number was counted at 24h, 48h and 72h after transfection. (B) Inhibition of miR-192 or miR-204promotes HCC cell growth. Cell number was counted at 24h, 48h and 72h after transfection. (C) Combined effects of miR-192/-204 with SAHA (suberoylanilidehydroxamic acid, also known as Vorinostat) on HCC viability. 20 nmol/L miRNA mimics or NC RNA was transfected into HCC cells treated with 10μmol/L SAHA. Cell number was counted at 24h, 48h and 72h after transfection. (D) Colony formation assays. At the 10th day after transfection, colony number in each well was counted. (E) Overexpression of miR-192, miR-204 or siHOTTIP-1 induces SMMC7721, HepG2 or Hep3B cell apoptosis. Apoptosis was determined with FACSCalibur flow cytometer at 72h after transfection. All results of the mean of triplicate assays with standard deviation of the mean are presented. *P< 0.05, **P< 0.01.

We found that suppressing HOTTIP by miR-192 and miR-204 can significantly reduce viability of SMMC7721,HepG2 and Hep3B cells (Fig 3A). Either miRNAs or HOTTIP siRNAs showed similar inhibition impact on SMMC7721 cell growth at 72h after RNA delivery (miR-192: 17.9%, miR-204: 20.5%, siHOTTIP-1: 23.1%, siHOTTIP-2: 23.1%; all *P*<0.05). Similar results were observed in HepG2 at 72h after transfection (miR-192: 36.4%, miR-204: 31.8%, siHOTTIP-1: 47.7%, siHOTTIP-2: 43.1%; all *P*<0.01). The suppressive effects of both miRNAs on HCC cell proliferation were also validated in Hep3B cell line (miR-192: 45.1%, miR-204: 37.8%, siHOTTIP-1: 43.9%, siHOTTIP-2: 51.2%; all *P*<0.01). Interestingly, there was a combined effect on inhibition of cell proliferation when miR-192 and miR-204 mimics were co-transfected into HCC cells (SMMC7721: 35.9%, HepG2: 54.5%, Hep3B: 60.9%; all *P*<0.01). When the pCMV-HOTTIP plasmid was transfected into HCC cells with miR-192, miR-204 or both miRNAs over-expression, suppressed HCC cell proliferation could be rescued(S1 Fig). After inhibitors of both miRNAs were delivered into HCC cells, cell proliferation was stimulated (Fig 3B). Antagonizing endogenousmiR-192 expression led to 23.7%,42.1% or34.6% increased SMMC7721, HepG2 orHep3B cell growth at 72h (all *P*<0.05). There was a 15.8%, 34.2% or 25.6% increase of SMMC7721, HepG2 or Hep3B proliferation after inhibition of endogenousmiR-204 expression(all *P*<0.05). As shown in Fig 3C, combined effects of miR-192/-204 with SAHA (suberoylanilidehydroxamic acid, also known as Vorinostat) on HCC viability were also observed.

Colony formation assays also support the tumor suppressor nature of both miRNAs (Fig 3D). Consistent with cell viability assays, miR-192 and miR-204, alone and in combination, are able to inhibit colony formation significantly (all *P*<0.05). The cell proliferation suppression via HOTTIP inhibition may be attributed to apoptosis. At 72h after transfection, HOTTIP siRNAs, miR-192 or miR-204 could induce significantly apoptosis (all *P*<0.05) (Fig 3E). However, both miRNAs cannot induce obvious cell cycle arrest (S2 Fig). We also examined impacts of miR-192/-204 on migration ability of HCC cells through wound healing assays. However, no difference in migratory behavior was observed in miRNA-treated cells versus controls (S3 Fig).

### MiR-192 and miR-204 modulate *GLS1* expression and GLS1-mediated cell growth control

**Fig 4:**
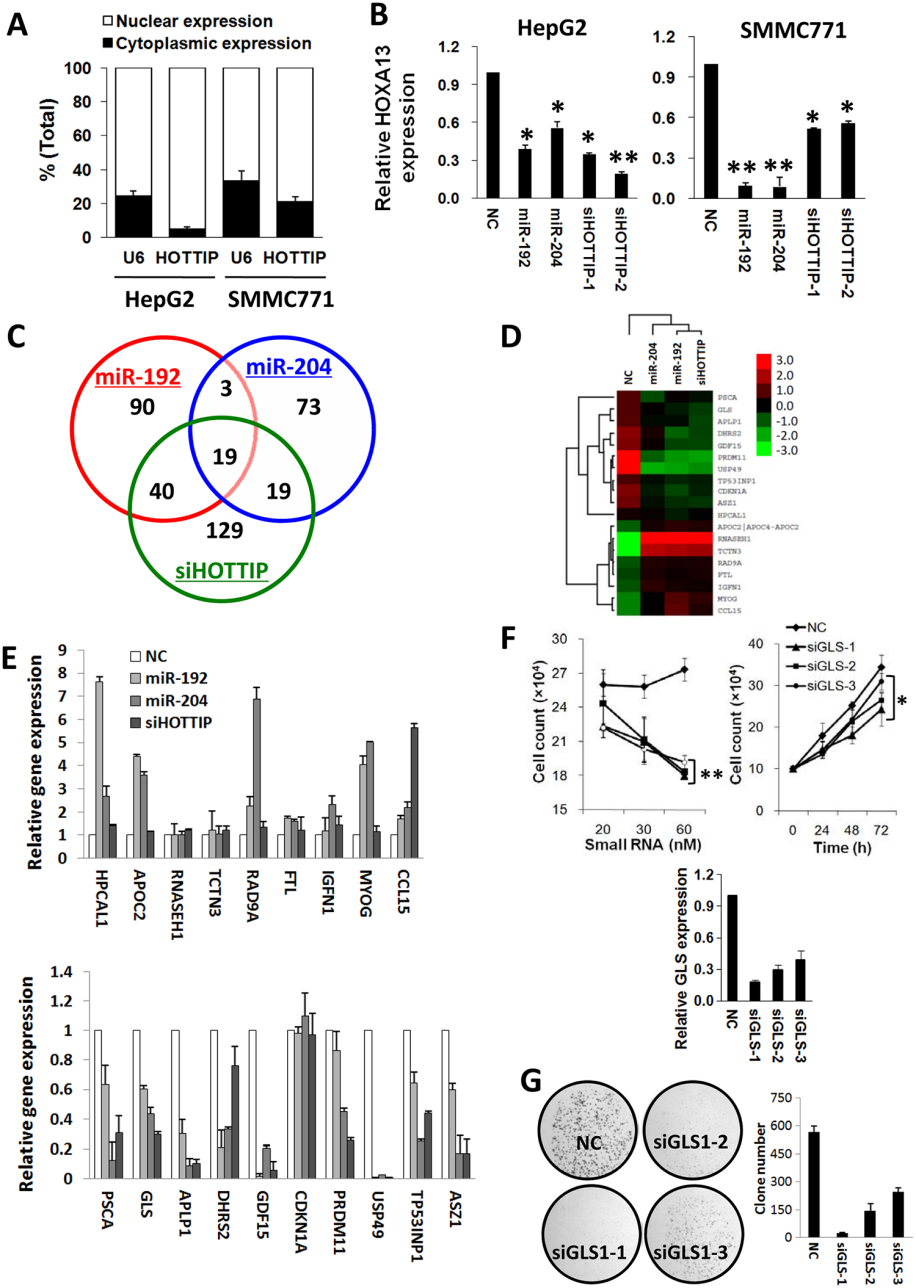
MiR-192 and miR-204 modulate expression of multiple HOTTIP downstream genes. (A) Cellular localization of lncRNA HOTTIP in HCC cells. (B)miR-192and miR-204 inhibit HOXA13 expression. 20 nmol/L miR-192 mimics, miR-204 mimics, siHOTTIP-1, siHOTTIP-2 or NC RNA were transfected into SMMC7721 and HepG2 cells. HOXA13 mRNA expression was detected using qRT-PCR. All data of HOXA13expression were normalized to *β-actin* or *GAPDH* mRNA expression levels. All results of the mean of triplicate assays with standard deviation of the mean are presented. *P< 0.05, **P< 0.01. (C) Deregulated genes in HepG2 cells after delivery of miR-192, miR-204 or siHOTTIP-1 using gene profiling arrays. (D) Cluster analysis of mRNAs expression profiles of the miR-192 group, the miR-204 group or the siHOTTIP-1 group versus the NC RNA group. Overexpression is coded in red, whereas under expression is indicated in green. (E) Candidate gene expression was validated using qRT-PCR. All data of gene expression were normalized to *β-actin* or *GAPDH* mRNA expression levels.(F) Inhibition ofGLS1 expression inhibits SMMC7721 cell growth. Up left panel, different doses of NC RNA or GLS1 siRNAs (20–60 nM) was transfected into SMMC7721 cells. Cell number was counted at 48 h after transfection. Up right panel, 30 nM NC RNA or GLS1 siRNA was transfected into SMMC7721 cells. Cell number was counted at 24, 48 or 72 h after transfection. Relative GLS1 gene expression was detected using qRT-PCR (low). (G) Colony formation assays. 30 nM NC RNA or GLS1 siRNA was transfected into SMMC7721 cells. After 10 days, colony number in each well was counted. *P< 0.05, **P< 0.01.

We firstly evaluated cellular localization of lncRNA HOTTIP in HCC cells. Cells were fractionated into nuclear and cytoplasmic fractions and RNA was extracted separately. HOTTIP was detected predominantly in the nuclear fraction as U6 RNA did in HCC cells (Fig 4A). Considering the exclusive localization of HOTTIP in the nuclei and AGO2 generally interacts with RNAs exported to cytoplasm, we examined if expression of 'nuclear' HOTTIP-lncRNA could be inhibited by miRNA-192/-204 via qRT-PCR using nuclear and cytoplasmic RNAs isolated from cells treated with miRNAs. As shown in Supplementary Figure 4, we found that miRNA-192/-204 could directly silence HOTTIP expression in either nucleus or cytoplasm. Considering *HOXA13*asone of 5’ end HOX A genes (known HOTTIP target genes), we then measured its expression in HCC cells. In line with previous studies, significantly down-regulated *HOXA13* mRNA expression in both HCC cell lines after silencing HOTTIP by either siRNAs or miR-192/-204(all *P*<0.05) (Fig 4B).

To further disclose the potential molecular mechanism how miRNAs influence HCC viability via suppressing lncRNA HOTTIP, we profiled whole genome mRNA expression of HepG2 cells transfected with NC RNA, miR-192, miR-204 or siHOTTIP-1 (Fig 4). Compared to NC RNA, there were 152, 114 or 207 differentially expressed genes caused by miR-192, miR-204 or siHOTTIP-1. Among these genes, a total of 19 genes were consistently de-regulated by any of these three small RNAs (Fig 4C and 4D). Gene Ontology analyses indicate that most genes are involved in positive or negative regulation of cell proliferation as well as cell death control (S2 Table), which is in agreement with the role of miR-192 and miR-204 on HCC cell proliferation.

We then validated microarray profiling identified 19 genes using qRT-PCR (Fig 4E). Among these successfully validated downstream genes, we chose *GLS1* as the candidate gene to investigate its considering its importance in glutaminolysis and tumorigenesis. As shown in S5 Fig, overexpression of lncRNA HOTTIP does enhance GLS expression in SMMC7721, HepG2 and Hep3B cells. Interestingly, we found that silencing its expression could significantly inhibit proliferation of HCC cells, in both a dose-dependent and time-dependent way (all *P*<0.05)(Fig 4F). We also examined impacts of *GLS1* suppressing oncolony formation of HCC cells, which also support the oncogenenature of *GLS1* (Fig 4G).

### Clinical significance of negative regulation of HOTTIP by miR-192 and miR-204

**Fig 5:**
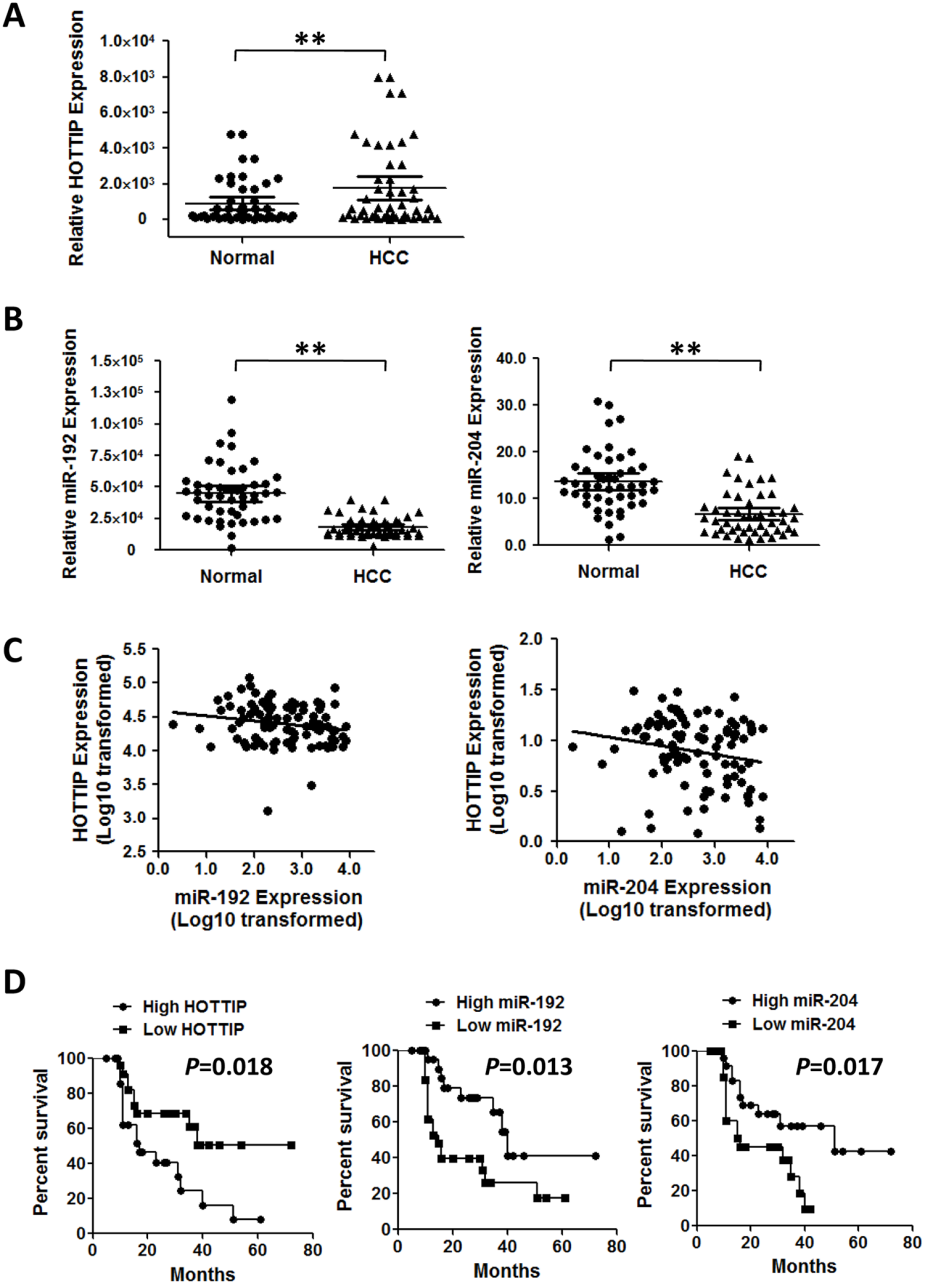
MiR-192 and miR-204 are negatively correlated with HOTTIP expression in HCC tissue specimens. (A) HOTTIP was quantified using qRT-PCR in 48 tumor-normal pairs. All data of HOTTIP expression were normalized to *β-actin* mRNA expression levels. (B) MiR-192 and miR-204 expression were measured in the same48 tumor-normal pairs. All miRNA expression data were normalized to U6 small RNA expression. (C) Correlations between miR-192 and HOTTIP or miR-204 and HOTTIP were presented. (D) Survival analyses of three ncRNAsin48 HCC patients. Survival curves were constructed using the Kaplan-Meier method and evaluated using the log-rank test. *P< 0.05, **P< 0.01.

To determine relationship between HOTTIP and miR-192/-204 *in vivo*, we detected expression of miR-192, miR-204 and HOTTIP in 48 tumor-normal pairs (S3 Table). There was significantly higher HOTTIP expression in HCC samples than normal specimens (median, 1.7×10^3^ versus 891.0, *P*<0.01)(Fig 5A). On the contrary, less miR-192 or miR-204 could be detected in HCC tissues compared to adjacent normal tissues (median, 1.8×10^4^ versus 4.5×10^4^ or, 6.7 versus 13.6; both *P*<0.01)(Fig 5B). We also found significant negative correlation between HOTTIP and miR-192 or miR-204 in tissues (Spearman’s correlation: HOTTIP versus miR-192: *r* = -0.2, *P*<0.05; HOTTIP versus miR-204: *r* = -0.2, *P*<0.05)(Fig 5C).

We then evaluate the impacts of miR-192, miR-204 and HOTTIP expression on HCC prognosis. HCC patients with high HOTTIP expression had much shorter overall survival time than those with low HOTTIP expression (median, 14.5 versus 28.5 months; *P* = 0.018)(Fig 5D). However, individuals with high miR-192 or miR-204 level showed better prognosis compared to ones with low expression of each miRNA on multivariate survival analysis (miR-192: median, 26.5 versus 14.0 months; *P* = 0.013; miR-204: 26.0 versus 15.0 months; *P* = 0.017)(Fig 5D).

### MiR-192 and miR-204 inhibit HCC growth *in vivo*

**Fig 6:**
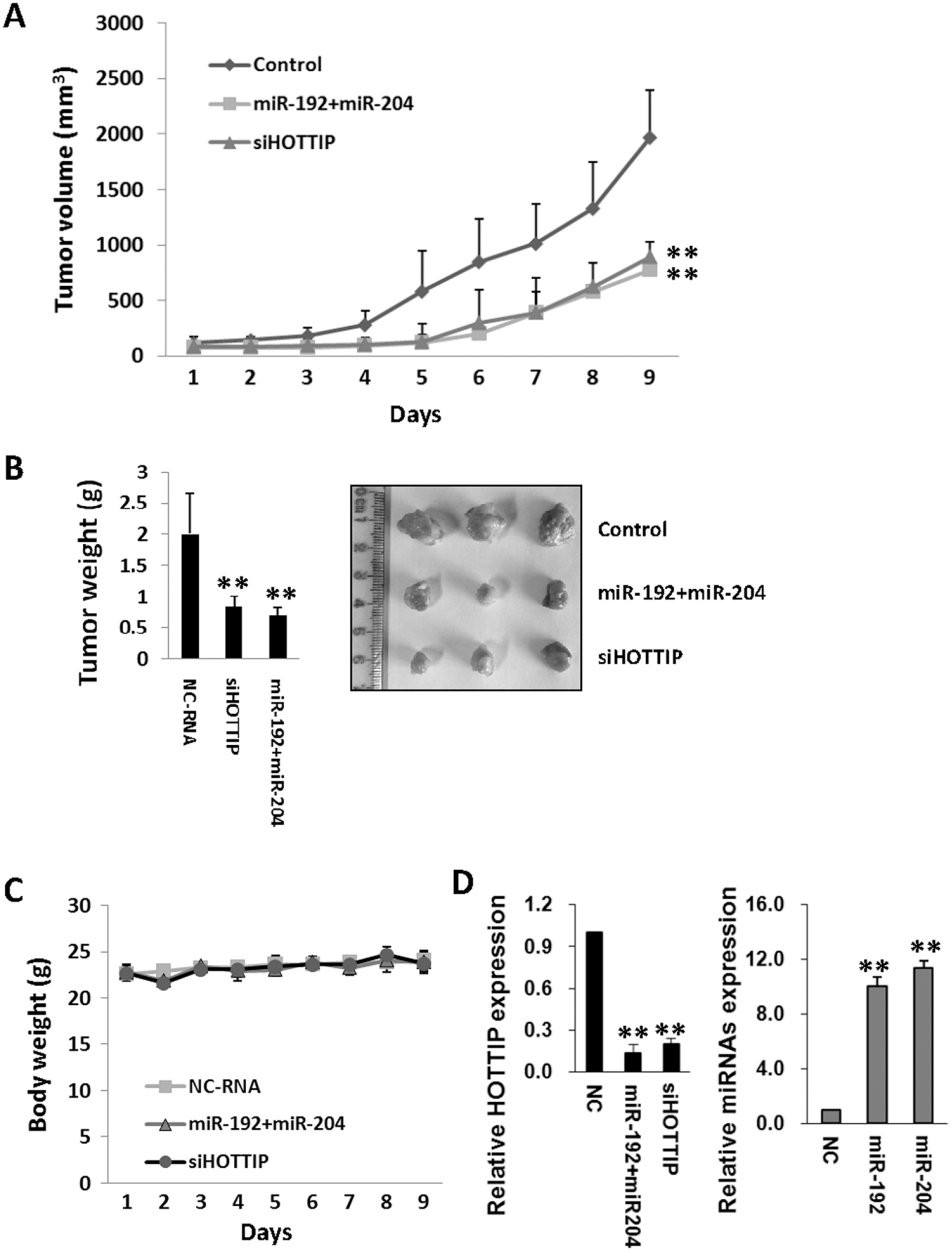
MiR-192 and miR-204 inhibit HCC growth *in vivo*. (A, B) the growth of tumors from both miR-192 and miR-204-up-regulated HepG2 xenografts or tumors from xenografts with stable transfection of HOTTIP siRNA constructs was inhibited significantly compared with that of tumors formed from control xenografts after 9 days. (C) There were no significant differences of mice weight between controls or miRNAs treated groups. (D) MiR-192 and miR-204 or HOTTIP siRNA inhibit HOTTIP expression in xenografts. Expression of HOTTIP and different miRNAs was detected by qRT-PCR. **P< 0.01.

We found that the growth of tumors from both miR-192 and miR-204 up-regulated HepG2 xenografts or xenografts with stable transfection of HOTTIP siRNA constructs was significantly inhibited compared with that of tumors formed from control xenografts after 9 days (Fig 6A and 6B). There were no significant differences of mice weight between controls or miRNAs treated groups (Fig 6C). qRT-PCR data showed that miR-192 and miR-204 or HOTTIP siRNA could significantly inhibit HOTTIP expression in xenografts (Fig 6D).

## Discussion

LncRNA HOTTIP is an important oncogene in HCC [6]. However, its post-transcriptional fine-regulation was still unclear. To the best of our knowledge, we here for the first time revealed that two miRNAs (miR-192 and miR-204) can suppress oncogene *HOTTIP* expression and HCC viability via the AGO2-mediated RNAi pathway. Consistent with this notion, a significant negative correlation between both miRNAs and HOTTIP exists in HCC tissue specimens. Interestingly, the dysregulation of the three ncRNAs was associated with overall survival of HCC patients, indicating their potential as prognostic markers. *In vivo* xenografts data highlight that this miRNA-mediated epigenetic regulation might be therapeutically relevant for HCC.

Mammalian miRNA target sites primarily locate in mRNA 3’-UTR [25,26],since active translation may interrupt miRNA binding within the protein-coding regions [27]. LncRNAs are more readily accessible to miRNAs as a whole because of no proteins translated. Consistent with this hypothesis, several lncRNAs, such asPTENP1, HULC, GAS5, loc285194,HOTAIR as well as HOTTIP, have been identified as miRNA targets in various cancers [7,22,23,28,29], which provide further layers of understanding lncRNA regulation during carcinogenesis.

It has been found that miR-192 and miR-204 function as tumor suppressors in multiple cancers including HCC. As one of the P53-inducable miRNAs [30,31], miR-192 takes part in cancer development and progression through targeting DHFR, TYMS, RB1, ZEB2, BCL2 and VEGFA [32–36]. Additionally, miR-192 is one of plasma miRNAs that provided a high diagnostic accuracy of early-stage HCC, indicating its clinical relevance [37]. Several target genes of miR-204, including HOXA10, MEIS1, FOXC1, MAP1LC3B, BCL2, NTRK2, SOX4 and EphB2, have been identified in different cancers [38–42]. However, the role of miR-204 in HCC is still unclear. We revealed that miR-204 suppresses oncogene *HOTTIP* expression in HCC, which is consistent with its tumor suppressor role in other malignancies. Although Quagliata et al found that HOTTIP and HOXA13 are involved in HCC development by associating their expression to metastasis and survival in HCC patients [6], they found that siRNA-HOTTIP treated cells do not display any significant impairment of their migratory behavior [6]. This is in line with our observations that miR-192/-204 did not influence HCC migration.

Significantly inhibited cell proliferation might be largely due to apoptosis after delivery of miR-192 or miR-204 into HCC cells. One possible explanation is that HOTTIP inhibition by miR-192 or miR-204 may lead to abnormal glutaminolysis and then apoptosis. Cancer cells tend to take up more glucose than most normal cells despite the availability of oxygen, which is called the Warburg effect [43]. Glutaminolysis is another hallmark of cancer cells in addition to aberrant glucose metabolism [44]. Mitochondrial GLS1 plays an essential part in glutaminolysis through catalyzing the conversion of glutamine to glutamate [45]. Glutamate is further catabolized in the Krebs cycle to produce ATP, nucleotides, certain amino acids, lipids, and glutathione [46]. Many cancer cell lines including HepG2 display addiction to glutamine and are sensitive to glutamine starvation or dysregulation of the glutaminolysis genes, such as *GLS1* [46,47]. Interestingly, down-regulated *GLS1*expression was observed after HCC cells transfected with miR-192, miR-204 or HOTTIP siRNA. Silencing GLS1 expression in HCC cells resulted in obviously retardant cell proliferation and colony formation. Therefore, we speculate that the miR-192/-204-HOTTIP axis may interrupt glutaminolysis of HCC and, thus, suppress cell viability.

Although Ago2 commonly locates in cytoplasm, Ago2 and RNAi factors Dicer, TRBP, and TRNC6A/GW182were also found in the human nucleus and can mediate functional RNAi in nucleus[48]. Moreover, mature miRNAs can be transported from cytoplasm to nucleus by importin 8[49]. That is, there are essential machinery for Ago2-miRNA mediated RNA silencing in human nucleus, which explained why HOTTIP mainly existing in the nuclei could physically interact with Ago2. Similar miRNAs regulation mechanisms were also observed in other nucleus lncRNAs. For instance, nucleus lncRNA MALAT1 is a well-known oncogene and could be directly regulated by several miRNAs [50–52].

In summary, we identified lncRNA HOTTIP as a novel target of miR-192 and miR-204. This posttranscriptional regulation showed significant impact on proliferation of HCC cells. The identification of *GLS1* as a potential downstream gene of the miR-192/-204-HOTTIP axis highlights the involvement of glutaminolysis in HCC. In view of our present results showing attenuated miR-192/-204 expression and enhanced HOTTIP expression in human HCC clinical specimens and their association with patient survival, we hypothesize that the miR-192/-204-HOTTIP axis may be an attractive target for prognostic and therapeutic interventions in HCC.

## Materials and Methods

### Ethics statement

For Human Subject Research, this study was approved by the Institutional Review Board of Huaian No. 2 Hospital (approval number: 20140305). Written informed consent was obtained from each subject at recruitment. For Animal Research, all experiments were performed according to the guidelines approved by the Institutional Review Board of Beijing Institute of Radiation Medicine for the care and use of laboratory animals (approval number: 14–51201).

### Cell culture and reagents

SMMC7721, HepG2 and Hep3B cells were cultured in RPMI-1640 or DMEM media with 10% fetal bovine serum (Hyclone) at 37°C with 5% CO_2_. All miRNA mimics (miR-138, miR-18, miR-192, miR-215, miR-19, miR-204 and miR-211), miRNA inhibitors(miR-192 and miR-204) and small interfering RNA (siRNA) duplexes (siHOTTIP-1 and siHOTTIP-2) were products of Genepharma (Shanghai, China). The negative control RNA duplex (NC) for miRNA mimics, miRNA inhibitors or siRNAs (Genepharma, Shanghai, China)was nonhomologous to any human genome sequence. All trasfections of small RNAs were done with Lipofectamine RNAi Max (Invitrogen).

### Quantitative reverse transcription PCR (qRT-PCR)

After isolation from culture cells or clinical tissues with Trizol reagent (Invitrogen), each RNA sample was treated with RNase-Free DNase to remove genomic DNA (Invitrogen). These RNA samples were then reverse transcribed into cDNAs using Revert Ace kit (TOYOBO, Osaka, Japan). Human miRNAs and U6 were detected with their specific stem-loop RT-PCR primers (Ribobio, Guangzhou, China) [53]. *HOTTIP*, *HOXA13*, and other potential HOTTIP downstream gene expression were measured through the SYBR-Green qRT-PCR. The expression of individual gene was calculated relative to the *β-actin* or *GAPDH* expression [53–55].

### RNA immunoprecipitation

The Imprint RNA Immunoprecipitation Kit (Sigma, St. Louis, USA) was used in RNA immunoprecipitation with the AGO2 antibody (Cell signaling, Rockford, USA). The AGO2 antibody was then recovered by protein A/G beads. HOTTIP, miR-192 and miR-204 RNA levels in the precipitates were measured by qRT-PCR.

### HOTTIP reporter constructs

The pGL3-Control plasmid (Promega, Madison, USA) was digested with *Xba*I (Promega). The long restricted DNA products were recovered and treated with Mung Bean Nuclease (Promega) to degrade single-stranded extensions from the ends of DNA and leave blunt ends. The sequence corresponding to the wild-type HOTTIP 3’end (1561-3840nt) was amplified with HepG2 cDNA using Pyrobest DNA Polymerase (TaKaRa). The PCR primer pair used was: 5’-AAGGCGGTTTTACATACTGGTC-3’/ 5’-TAGCACCTGTAGTTGCCCATTCC-3’. The PCR products with blunt ends were ligated into the appropriately digested pGL3-Control (Promega) containing the firefly luciferase gene as a reporter. The resultant plasmid, designated pGL3-HOTTIP, was sequenced to confirm the orientation and integrity. The *HOTTIP* reporter gene plasmid with mutant miR-192 binding site or mutant miR-204 binding site was constructed with QuikChange Site-Directed Mutagenesis kit (Stratagene, La Jolla, CA). These mutant plasmids were confirmed by DNA sequencing and named as pGL3-Mut192 or pGL3-Mut204.

### Dual luciferase reporter assays

SMMC7721 and HepG2 cells (6×10^4^) were seeded in 24-well plates and transfected with both 50 ng of reporter constructs (pGL3-Control, pGL3-HOTTIP, pGL3-Mut192 or pGL3-Mut204) plus 20 nmol/L small RNAs (miR-192 mimics, miR-204 mimics or NC RNAs) using Lipofectamine 2000 (Invitrogen) when grown to 50% confluence. To standardize transfection efficiency, pRL-SV40 (1 ng) (Promega) containing renilla reniformis luciferase was cotransfected. Both firefly luciferase activity and renilla luciferase activity were detected at 48h after transfection using a luciferase assay system (Promega). For each luciferase construct, three independent transfections were done (each in triplicate). Fold increase was calculated by defining the activity of the pGL3-Control vector as 1.

### Cell proliferation, cell cycle and apoptosis analyses

SMMC7721, HepG2 and Hep3B cells (1×10^5^) were seeded in 12-well plates and transfected with 20 nmol/L miR-192 mimics, miR-204 mimics, HOTTIP siRNAs (siHOTTIP-1 and siHOTTIP-2), miR-192 inhibitors, miR-204 inhibitors or NC RNA (Genepharma), respectively. Cells were then harvested by trypsin digestion, washed by cold PBS twice, dyed with trypan blue and counted under microscopy at 24h, 48h and 72h after transfection. Cells were transfected with 20nmol/L miR-192 mimics, miR-204 mimics, or NC and harvested 48h after transfection. After washing with cold PBS twice, cells were fixed with ethanol at -20°C overnight and washed with cold PBS twice again. After treated with RNase A at 37°C for 0.5h and dyed with PI, the samples were detected with the FACSCalibur flow cytometer (FCM) (BD Biosciences). During apoptosis assays, nonadherent and adherent cells were collected at 72h after transfection. Apoptosis was determined using the Alexa Fluor 488 annexin V/Dead Cell Apoptosis Kit (Invitrogen) with the FACSCalibur FCM.

### Colony formation assays

A total of 1,500 SMMC7721 or HepG2 cells were seeded into a 6-well cell culture plate and transfected with 20 nmol/L miR-192 mimics, miR-204 mimics, siHOTTIP-1, or NC RNA, respectively. 10 nmol/L miR-192 mimics and 10 nmol/L miR-204 mimics were co-transfected into HCC cells. After 10 days, cells were washed with cold PBS twice and fixed with 3.7% formaldehyde. After cells were dyed with crystal violet, colony number in each well was counted.

### Wound healing assays

When the cell layer reached ~90% confluence, a wound was scratched by a 10μl pipette tip. Cells were then cultured at 37°C for another 48h. The average extent of wound closure was quantified.

### Subcellular fractionation

The cytosolic and nuclear fractions of SMMC7721or HepG2 cells were collected using the nuclear/cytoplasmic Isolation Kit (Biovision, San Francisco, CA). We performed the detailed experimental procedures according to the manufacturer’s instructions.

### Gene expression profiling

Total RNA from HepG2 cells transfected with 20 nmol/L NC RNA, miR-192 mimics, miR-204 mimics or siHOTTIP-1 was extracted. Whole genome gene expression of these samples was measured using OneArray Plus chips (Phalanx Biotech Group). Differential expressed genes between the NC RNA group and the miR-192 group, the NC RNA group and the miR-204 group, or the NC RNA group and the siHOTTIP-1 group were identified separately. The differentially expressed genes were identified following the criteria log2 (Fold change) ≥ 0.585 and *P*<0.05. The microarray data have been deposited at the National Center for Biotechnology Institute Gene Expression Omnibus (GEO) repository under accession number GSE60912.

### Patients and tissue specimens

There were a total of 48 HCC patients recruited in the current study. All patients received curative resection for HCC in Huaian No. 2 Hospital (Huaian, Jiangsu Providence, China) between February 2008 and December 2012. Prior to the surgery, no patients received any local or systemic anticancer treatments. All patients were postoperatively followed after surgery until May 2014, with a median follow-up of 18 months(range, 5–72 months). The relevant clinic-pathological characteristics of the studied subjects are shown in S5 Fig. This study was approved by the Institutional Review Board of Huaian No. 2 Hospital. At recruitment, written informed consent was obtained from each subject.

### HCC xenograft

To evaluate the tumor suppressor role of miR-192 and miR-204 *in vivo*, we firstly cloned miR-192andmiR-204 mature sequence or siHOTTIP sequence after the CMV promoter in a tandem manner into pcDNA3.1 vector. The plasmids were named as pcDNA3.1-miR-192-miR-204 or pcDNA3.1-siHOTTIP and transfected into HepG2 cells. After G418 (Geneticin) selection, we isolated a stable cell clone with relative high expression of miR-192 and miR-204 and another stable cell clone with significant silencing of lncRNA HOTTIP. Five-week-old female nude BALB/c mice were purchased from Vital River Laboratory (Beijing, China).1×10^8^HepG2 cells with stable transfection of pcDNA3.1-miR-192-miR-204, pcDNA3.1-siHOTTIPor pcDNA3.1 vector were inoculated subcutaneously into fossa axillaris of 6 nude mice (*n* = 3 per group). Tumor volumes were measured every day after tumor volumes equaled to or were greater than 80 mm^3^. All procedures involving mice were approved by the institutional Review Board of Beijing Institute of Radiation Medicine.

### Statistics

The difference between two groups was calculated using Student’s *t* test. One-way analysis of variance was used to comparing differences between three or more groups. Spearman’s correlation was used to test the significance of association between miR-192 or miR-204 expression and HOTTIP expression. Association between miRNAs or HOTTIP and overall survival was examined using Kaplan-Meier plots and Cox proportional hazard regression analyses. A *P* value of less than 0.05 was used as the criterion of statistical significance. All analyses were performed with SPSS software package (Version 16.0, SPSS Inc.) or GraphPad Prism (Version5, GraphPad Software, Inc.).

## Supporting Information

S1 Table MiRcode prediction of miRNA potentially targeting lncRNA HOTTIP. (DOCX)

S2 Table Gene Ontolog analyses with 19 differentially expressed genes. (DOCX)

S3 Table The relevant clinic-pathological characteristics of HCC cases. (DOCX)

S1 Fig Rescue assays. A. Rescue assays in SMMC7721 cells. B. Rescue assays in HepG2 cells.
(TIF)

S2 Fig Cell cycle analyses. A. Cell cycle analyses in SMMC7721 cells. B. Cell cycle analyses in HepG2 cells.
(TIFF)

S3 Fig Wound healing assays. A. Wound healing assays in SMMC7721 cells. B. Wound healing assays in HepG2 cells.
(TIFF)

S4 Fig MiRNA-192/-204 could directly silence HOTTIP expression in either nucleus or cytoplasm in SMMC7721 cells (A) or HepG2 cells (B). (TIFF)

S5 Fig Overexpression HOTTIP induces GLS1 expression in HCC cells. A. GLS1 expression in HCC cells. B. HOTTIP expression in HCC cells.
(TIFF)
