# Correction: fMiRNA-192 and miRNA-204 Directly Suppress lncRNA HOTTIP and Interrupt *GLS1*-Mediated Glutaminolysis in Hepatocellular Carcinoma

**DOI:** 10.1371/journal.pgen.1005825

**Published:** 2016-01-25

**Authors:** The PLOS Genetics Staff

In the title of this article, “fMiRNA-192” should be “MiRNA-192.”

The correct title is: “MiRNA-192 and miRNA-204 Directly Suppress lncRNA HOTTIP and Interrupt *GLS1*-Mediated Glutaminolysis in Hepatocellular Carcinoma”.

The correct citation is: Ge Y, Yan X, Jin Y, Yang X, Yu X, Zhou L, et al. (2015) MiRNA-192 and miRNA-204 Directly Suppress lncRNA HOTTIP and Interrupt *GLS1*-Mediated Glutaminolysis in Hepatocellular Carcinoma. PLoS Genet 11(12): e1005726. doi:10.1371/journal.pgen.1005726

The publisher apologizes for this error.
