# Supplementary material for: fMiRNA-192 and miRNA-204 Directly Suppress lncRNA HOTTIP and Interrupt GLS1-Mediated Glutaminolysis in Hepatocellular Carcinoma
Source: PLoS Genet. 2015 Dec 28;11(12):e1005726. doi: 10.1371/journal.pgen.1005726 (PMC4692503; doi:10.1371/journal.pgen.1005726)
Supplement: S1 Table — (DOCX) [file pgen.1005726.s001.docx]

**Supplementary Table 1**. MiRcode prediction of miRNA potentially targeting lncRNA HOTTIP

| **microRNA family** | **Seed position** | **Seed type** | **Transcript region** | **Repeat** | **Conservation** | | |
| --- | --- | --- | --- | --- | --- | --- | --- |
|  |  |  |  |  | **Primates** | **Mammals** | **Other vert.** |
| miR-130ac/301ab/301b/301b-3p/454/721/4295/3666 | [**chr7:27246744**](http://genome.ucsc.edu/cgi-bin/hgTracks?db=hg19&wgEncodeGencodeSuper=show&wgEncodeGencodeV12ViewGenes=full&wgEncodeGencodeBasicV12_sel=0&wgEncodeGencodeCompV12_sel=1&wgEncodeGencodePseudoGeneV12_sel=1&position=chr7:27246695-27246794&hgt.customText=http://www.mircode.org/mircode/gencode_mirsites_highconsfamilies.bed) | 7-mer-m8 | ncRNA | no | 56 % | 0 % | 0 % |
| miR-132/212/212-3p | [**chr7:27240450**](http://genome.ucsc.edu/cgi-bin/hgTracks?db=hg19&wgEncodeGencodeSuper=show&wgEncodeGencodeV12ViewGenes=full&wgEncodeGencodeBasicV12_sel=0&wgEncodeGencodeCompV12_sel=1&wgEncodeGencodePseudoGeneV12_sel=1&position=chr7:27240401-27240500&hgt.customText=http://www.mircode.org/mircode/gencode_mirsites_highconsfamilies.bed) | 7-mer-A1 | ncRNA | no | 67 % | 0 % | 0 % |
| miR-93/93a/105/106a/291a-3p/294/295/302abcde/372/373/428/519a/520be/520acd-3p/1378/1420ac | [**chr7:27245734**](http://genome.ucsc.edu/cgi-bin/hgTracks?db=hg19&wgEncodeGencodeSuper=show&wgEncodeGencodeV12ViewGenes=full&wgEncodeGencodeBasicV12_sel=0&wgEncodeGencodeCompV12_sel=1&wgEncodeGencodePseudoGeneV12_sel=1&position=chr7:27245685-27245784&hgt.customText=http://www.mircode.org/mircode/gencode_mirsites_highconsfamilies.bed) | 7-mer-m8 | ncRNA | no | 11 % | 0 % | 0 % |
| miR-137/137ab | [**chr7:27246824**](http://genome.ucsc.edu/cgi-bin/hgTracks?db=hg19&wgEncodeGencodeSuper=show&wgEncodeGencodeV12ViewGenes=full&wgEncodeGencodeBasicV12_sel=0&wgEncodeGencodeCompV12_sel=1&wgEncodeGencodePseudoGeneV12_sel=1&position=chr7:27246775-27246874&hgt.customText=http://www.mircode.org/mircode/gencode_mirsites_highconsfamilies.bed) | 8-mer | ncRNA | no | 78 % | 4 % | 0 % |
| miR-138/138ab | [**chr7:27241450**](http://genome.ucsc.edu/cgi-bin/hgTracks?db=hg19&wgEncodeGencodeSuper=show&wgEncodeGencodeV12ViewGenes=full&wgEncodeGencodeBasicV12_sel=0&wgEncodeGencodeCompV12_sel=1&wgEncodeGencodePseudoGeneV12_sel=1&position=chr7:27241401-27241500&hgt.customText=http://www.mircode.org/mircode/gencode_mirsites_highconsfamilies.bed) | 7-mer-m8 | ncRNA | no | 67 % | 0 % | 0 % |
| miR-138/138ab | [**chr7:27245289**](http://genome.ucsc.edu/cgi-bin/hgTracks?db=hg19&wgEncodeGencodeSuper=show&wgEncodeGencodeV12ViewGenes=full&wgEncodeGencodeBasicV12_sel=0&wgEncodeGencodeCompV12_sel=1&wgEncodeGencodePseudoGeneV12_sel=1&position=chr7:27245240-27245339&hgt.customText=http://www.mircode.org/mircode/gencode_mirsites_highconsfamilies.bed) | 7-mer-m8 | ncRNA | no | 89 % | 30 % | 0 % |
| miR-141/200a | [**chr7:27246242**](http://genome.ucsc.edu/cgi-bin/hgTracks?db=hg19&wgEncodeGencodeSuper=show&wgEncodeGencodeV12ViewGenes=full&wgEncodeGencodeBasicV12_sel=0&wgEncodeGencodeCompV12_sel=1&wgEncodeGencodePseudoGeneV12_sel=1&position=chr7:27246193-27246292&hgt.customText=http://www.mircode.org/mircode/gencode_mirsites_highconsfamilies.bed) | 7-mer-m8 | ncRNA | yes | 0 % | 0 % | 0 % |
| miR-143/1721/4770 | [**chr7:27242306**](http://genome.ucsc.edu/cgi-bin/hgTracks?db=hg19&wgEncodeGencodeSuper=show&wgEncodeGencodeV12ViewGenes=full&wgEncodeGencodeBasicV12_sel=0&wgEncodeGencodeCompV12_sel=1&wgEncodeGencodePseudoGeneV12_sel=1&position=chr7:27242257-27242356&hgt.customText=http://www.mircode.org/mircode/gencode_mirsites_highconsfamilies.bed) | 7-mer-A1 | ncRNA | no | 67 % | 26 % | 0 % |
| miR-148ab-3p/152 | [**chr7:27246745**](http://genome.ucsc.edu/cgi-bin/hgTracks?db=hg19&wgEncodeGencodeSuper=show&wgEncodeGencodeV12ViewGenes=full&wgEncodeGencodeBasicV12_sel=0&wgEncodeGencodeCompV12_sel=1&wgEncodeGencodePseudoGeneV12_sel=1&position=chr7:27246696-27246795&hgt.customText=http://www.mircode.org/mircode/gencode_mirsites_highconsfamilies.bed) | 8-mer | ncRNA | no | 56 % | 0 % | 0 % |
| miR-150/5127 | [**chr7:27240974**](http://genome.ucsc.edu/cgi-bin/hgTracks?db=hg19&wgEncodeGencodeSuper=show&wgEncodeGencodeV12ViewGenes=full&wgEncodeGencodeBasicV12_sel=0&wgEncodeGencodeCompV12_sel=1&wgEncodeGencodePseudoGeneV12_sel=1&position=chr7:27240925-27241024&hgt.customText=http://www.mircode.org/mircode/gencode_mirsites_highconsfamilies.bed) | 7-mer-m8 | ncRNA | no | 44 % | 0 % | 0 % |
| miR-150/5127 | [**chr7:27245933**](http://genome.ucsc.edu/cgi-bin/hgTracks?db=hg19&wgEncodeGencodeSuper=show&wgEncodeGencodeV12ViewGenes=full&wgEncodeGencodeBasicV12_sel=0&wgEncodeGencodeCompV12_sel=1&wgEncodeGencodePseudoGeneV12_sel=1&position=chr7:27245884-27245983&hgt.customText=http://www.mircode.org/mircode/gencode_mirsites_highconsfamilies.bed) | 8-mer | ncRNA | no | 56 % | 0 % | 0 % |
| miR-15abc/16/16abc/195/322/424/497/1907 | [**chr7:27240355**](http://genome.ucsc.edu/cgi-bin/hgTracks?db=hg19&wgEncodeGencodeSuper=show&wgEncodeGencodeV12ViewGenes=full&wgEncodeGencodeBasicV12_sel=0&wgEncodeGencodeCompV12_sel=1&wgEncodeGencodePseudoGeneV12_sel=1&position=chr7:27240306-27240405&hgt.customText=http://www.mircode.org/mircode/gencode_mirsites_highconsfamilies.bed) | 7-mer-m8 | ncRNA | no | 78 % | 17 % | 0 % |
| miR-15abc/16/16abc/195/322/424/497/1907 | [**chr7:27241063**](http://genome.ucsc.edu/cgi-bin/hgTracks?db=hg19&wgEncodeGencodeSuper=show&wgEncodeGencodeV12ViewGenes=full&wgEncodeGencodeBasicV12_sel=0&wgEncodeGencodeCompV12_sel=1&wgEncodeGencodePseudoGeneV12_sel=1&position=chr7:27241014-27241113&hgt.customText=http://www.mircode.org/mircode/gencode_mirsites_highconsfamilies.bed) | 7-mer-m8 | ncRNA | no | 56 % | 9 % | 0 % |
| miR-17/17-5p/20ab/20b-5p/93/106ab/427/518a-3p/519d | [**chr7:27245364**](http://genome.ucsc.edu/cgi-bin/hgTracks?db=hg19&wgEncodeGencodeSuper=show&wgEncodeGencodeV12ViewGenes=full&wgEncodeGencodeBasicV12_sel=0&wgEncodeGencodeCompV12_sel=1&wgEncodeGencodePseudoGeneV12_sel=1&position=chr7:27245315-27245414&hgt.customText=http://www.mircode.org/mircode/gencode_mirsites_highconsfamilies.bed) | 8-mer | ncRNA | no | 22 % | 4 % | 0 % |
| miR-17/17-5p/20ab/20b-5p/93/106ab/427/518a-3p/519d | [**chr7:27245735**](http://genome.ucsc.edu/cgi-bin/hgTracks?db=hg19&wgEncodeGencodeSuper=show&wgEncodeGencodeV12ViewGenes=full&wgEncodeGencodeBasicV12_sel=0&wgEncodeGencodeCompV12_sel=1&wgEncodeGencodePseudoGeneV12_sel=1&position=chr7:27245686-27245785&hgt.customText=http://www.mircode.org/mircode/gencode_mirsites_highconsfamilies.bed) | 7-mer-m8 | ncRNA | no | 11 % | 0 % | 0 % |
| miR-181abcd/4262 | [**chr7:27245449**](http://genome.ucsc.edu/cgi-bin/hgTracks?db=hg19&wgEncodeGencodeSuper=show&wgEncodeGencodeV12ViewGenes=full&wgEncodeGencodeBasicV12_sel=0&wgEncodeGencodeCompV12_sel=1&wgEncodeGencodePseudoGeneV12_sel=1&position=chr7:27245400-27245499&hgt.customText=http://www.mircode.org/mircode/gencode_mirsites_highconsfamilies.bed) | 7-mer-m8 | ncRNA | no | 67 % | 9 % | 0 % |
| miR-184 | [**chr7:27244569**](http://genome.ucsc.edu/cgi-bin/hgTracks?db=hg19&wgEncodeGencodeSuper=show&wgEncodeGencodeV12ViewGenes=full&wgEncodeGencodeBasicV12_sel=0&wgEncodeGencodeCompV12_sel=1&wgEncodeGencodePseudoGeneV12_sel=1&position=chr7:27244520-27244619&hgt.customText=http://www.mircode.org/mircode/gencode_mirsites_highconsfamilies.bed) | 7-mer-m8 | ncRNA | no | 0 % | 0 % | 0 % |
| let-7/98/4458/4500 | [**chr7:27246609**](http://genome.ucsc.edu/cgi-bin/hgTracks?db=hg19&wgEncodeGencodeSuper=show&wgEncodeGencodeV12ViewGenes=full&wgEncodeGencodeBasicV12_sel=0&wgEncodeGencodeCompV12_sel=1&wgEncodeGencodePseudoGeneV12_sel=1&position=chr7:27246560-27246659&hgt.customText=http://www.mircode.org/mircode/gencode_mirsites_highconsfamilies.bed) | 7-mer-A1 | ncRNA | no | 44 % | 0 % | 0 % |
| miR-187 | [**chr7:27241730**](http://genome.ucsc.edu/cgi-bin/hgTracks?db=hg19&wgEncodeGencodeSuper=show&wgEncodeGencodeV12ViewGenes=full&wgEncodeGencodeBasicV12_sel=0&wgEncodeGencodeCompV12_sel=1&wgEncodeGencodePseudoGeneV12_sel=1&position=chr7:27241681-27241780&hgt.customText=http://www.mircode.org/mircode/gencode_mirsites_highconsfamilies.bed) | 7-mer-m8 | ncRNA | no | 78 % | 48 % | 23 % |
| miR-18ab/4735-3p | [**chr7:27238346**](http://genome.ucsc.edu/cgi-bin/hgTracks?db=hg19&wgEncodeGencodeSuper=show&wgEncodeGencodeV12ViewGenes=full&wgEncodeGencodeBasicV12_sel=0&wgEncodeGencodeCompV12_sel=1&wgEncodeGencodePseudoGeneV12_sel=1&position=chr7:27238297-27238396&hgt.customText=http://www.mircode.org/mircode/gencode_mirsites_highconsfamilies.bed) | 7-mer-A1 | ncRNA | no | 89 % | 30 % | 0 % |
| miR-18ab/4735-3p | [**chr7:27242389**](http://genome.ucsc.edu/cgi-bin/hgTracks?db=hg19&wgEncodeGencodeSuper=show&wgEncodeGencodeV12ViewGenes=full&wgEncodeGencodeBasicV12_sel=0&wgEncodeGencodeCompV12_sel=1&wgEncodeGencodePseudoGeneV12_sel=1&position=chr7:27242340-27242439&hgt.customText=http://www.mircode.org/mircode/gencode_mirsites_highconsfamilies.bed) | 8-mer | ncRNA | no | 67 % | 0 % | 0 % |
| miR-192/215 | [**chr7:27241747**](http://genome.ucsc.edu/cgi-bin/hgTracks?db=hg19&wgEncodeGencodeSuper=show&wgEncodeGencodeV12ViewGenes=full&wgEncodeGencodeBasicV12_sel=0&wgEncodeGencodeCompV12_sel=1&wgEncodeGencodePseudoGeneV12_sel=1&position=chr7:27241698-27241797&hgt.customText=http://www.mircode.org/mircode/gencode_mirsites_highconsfamilies.bed) | 7-mer-A1 | ncRNA | no | 89 % | 91 % | 77 % |
| miR-193/193b/193a-3p | [**chr7:27240664**](http://genome.ucsc.edu/cgi-bin/hgTracks?db=hg19&wgEncodeGencodeSuper=show&wgEncodeGencodeV12ViewGenes=full&wgEncodeGencodeBasicV12_sel=0&wgEncodeGencodeCompV12_sel=1&wgEncodeGencodePseudoGeneV12_sel=1&position=chr7:27240615-27240714&hgt.customText=http://www.mircode.org/mircode/gencode_mirsites_highconsfamilies.bed) | 7-mer-A1 | ncRNA | no | 33 % | 0 % | 0 % |
| miR-194 | [**chr7:27241595**](http://genome.ucsc.edu/cgi-bin/hgTracks?db=hg19&wgEncodeGencodeSuper=show&wgEncodeGencodeV12ViewGenes=full&wgEncodeGencodeBasicV12_sel=0&wgEncodeGencodeCompV12_sel=1&wgEncodeGencodePseudoGeneV12_sel=1&position=chr7:27241546-27241645&hgt.customText=http://www.mircode.org/mircode/gencode_mirsites_highconsfamilies.bed) | 7-mer-m8 | ncRNA | no | 44 % | 0 % | 0 % |
| miR-199ab-5p | [**chr7:27240400**](http://genome.ucsc.edu/cgi-bin/hgTracks?db=hg19&wgEncodeGencodeSuper=show&wgEncodeGencodeV12ViewGenes=full&wgEncodeGencodeBasicV12_sel=0&wgEncodeGencodeCompV12_sel=1&wgEncodeGencodePseudoGeneV12_sel=1&position=chr7:27240351-27240450&hgt.customText=http://www.mircode.org/mircode/gencode_mirsites_highconsfamilies.bed) | 7-mer-A1 | ncRNA | no | 56 % | 0 % | 0 % |
| miR-199ab-5p | [**chr7:27244686**](http://genome.ucsc.edu/cgi-bin/hgTracks?db=hg19&wgEncodeGencodeSuper=show&wgEncodeGencodeV12ViewGenes=full&wgEncodeGencodeBasicV12_sel=0&wgEncodeGencodeCompV12_sel=1&wgEncodeGencodePseudoGeneV12_sel=1&position=chr7:27244637-27244736&hgt.customText=http://www.mircode.org/mircode/gencode_mirsites_highconsfamilies.bed) | 7-mer-A1 | ncRNA | no | 56 % | 9 % | 0 % |
| miR-19ab | [**chr7:27241254**](http://genome.ucsc.edu/cgi-bin/hgTracks?db=hg19&wgEncodeGencodeSuper=show&wgEncodeGencodeV12ViewGenes=full&wgEncodeGencodeBasicV12_sel=0&wgEncodeGencodeCompV12_sel=1&wgEncodeGencodePseudoGeneV12_sel=1&position=chr7:27241205-27241304&hgt.customText=http://www.mircode.org/mircode/gencode_mirsites_highconsfamilies.bed) | 7-mer-A1 | ncRNA | no | 56 % | 0 % | 0 % |
| miR-19ab | [**chr7:27245115**](http://genome.ucsc.edu/cgi-bin/hgTracks?db=hg19&wgEncodeGencodeSuper=show&wgEncodeGencodeV12ViewGenes=full&wgEncodeGencodeBasicV12_sel=0&wgEncodeGencodeCompV12_sel=1&wgEncodeGencodePseudoGeneV12_sel=1&position=chr7:27245066-27245165&hgt.customText=http://www.mircode.org/mircode/gencode_mirsites_highconsfamilies.bed) | 7-mer-m8 | ncRNA | no | 100 % | 0 % | 0 % |
| miR-19ab | [**chr7:27246743**](http://genome.ucsc.edu/cgi-bin/hgTracks?db=hg19&wgEncodeGencodeSuper=show&wgEncodeGencodeV12ViewGenes=full&wgEncodeGencodeBasicV12_sel=0&wgEncodeGencodeCompV12_sel=1&wgEncodeGencodePseudoGeneV12_sel=1&position=chr7:27246694-27246793&hgt.customText=http://www.mircode.org/mircode/gencode_mirsites_highconsfamilies.bed) | 7-mer-m8 | ncRNA | no | 56 % | 0 % | 0 % |
| miR-1ab/206/613 | [**chr7:27240395**](http://genome.ucsc.edu/cgi-bin/hgTracks?db=hg19&wgEncodeGencodeSuper=show&wgEncodeGencodeV12ViewGenes=full&wgEncodeGencodeBasicV12_sel=0&wgEncodeGencodeCompV12_sel=1&wgEncodeGencodePseudoGeneV12_sel=1&position=chr7:27240346-27240445&hgt.customText=http://www.mircode.org/mircode/gencode_mirsites_highconsfamilies.bed) | 8-mer | ncRNA | no | 78 % | 9 % | 0 % |
| miR-203 | [**chr7:27244472**](http://genome.ucsc.edu/cgi-bin/hgTracks?db=hg19&wgEncodeGencodeSuper=show&wgEncodeGencodeV12ViewGenes=full&wgEncodeGencodeBasicV12_sel=0&wgEncodeGencodeCompV12_sel=1&wgEncodeGencodePseudoGeneV12_sel=1&position=chr7:27244423-27244522&hgt.customText=http://www.mircode.org/mircode/gencode_mirsites_highconsfamilies.bed) | 7-mer-m8 | ncRNA | no | 56 % | 0 % | 0 % |
| miR-203 | [**chr7:27246313**](http://genome.ucsc.edu/cgi-bin/hgTracks?db=hg19&wgEncodeGencodeSuper=show&wgEncodeGencodeV12ViewGenes=full&wgEncodeGencodeBasicV12_sel=0&wgEncodeGencodeCompV12_sel=1&wgEncodeGencodePseudoGeneV12_sel=1&position=chr7:27246264-27246363&hgt.customText=http://www.mircode.org/mircode/gencode_mirsites_highconsfamilies.bed) | 7-mer-m8 | ncRNA | yes | 56 % | 0 % | 0 % |
| miR-203 | [**chr7:27246332**](http://genome.ucsc.edu/cgi-bin/hgTracks?db=hg19&wgEncodeGencodeSuper=show&wgEncodeGencodeV12ViewGenes=full&wgEncodeGencodeBasicV12_sel=0&wgEncodeGencodeCompV12_sel=1&wgEncodeGencodePseudoGeneV12_sel=1&position=chr7:27246283-27246382&hgt.customText=http://www.mircode.org/mircode/gencode_mirsites_highconsfamilies.bed) | 7-mer-A1 | ncRNA | no | 22 % | 4 % | 0 % |
| miR-203 | [**chr7:27246350**](http://genome.ucsc.edu/cgi-bin/hgTracks?db=hg19&wgEncodeGencodeSuper=show&wgEncodeGencodeV12ViewGenes=full&wgEncodeGencodeBasicV12_sel=0&wgEncodeGencodeCompV12_sel=1&wgEncodeGencodePseudoGeneV12_sel=1&position=chr7:27246301-27246400&hgt.customText=http://www.mircode.org/mircode/gencode_mirsites_highconsfamilies.bed) | 7-mer-A1 | ncRNA | no | 67 % | 52 % | 0 % |
| miR-203 | [**chr7:27246677**](http://genome.ucsc.edu/cgi-bin/hgTracks?db=hg19&wgEncodeGencodeSuper=show&wgEncodeGencodeV12ViewGenes=full&wgEncodeGencodeBasicV12_sel=0&wgEncodeGencodeCompV12_sel=1&wgEncodeGencodePseudoGeneV12_sel=1&position=chr7:27246628-27246727&hgt.customText=http://www.mircode.org/mircode/gencode_mirsites_highconsfamilies.bed) | 7-mer-m8 | ncRNA | no | 67 % | 35 % | 0 % |
| miR-204/204b/211 | [**chr7:27245995**](http://genome.ucsc.edu/cgi-bin/hgTracks?db=hg19&wgEncodeGencodeSuper=show&wgEncodeGencodeV12ViewGenes=full&wgEncodeGencodeBasicV12_sel=0&wgEncodeGencodeCompV12_sel=1&wgEncodeGencodePseudoGeneV12_sel=1&position=chr7:27245946-27246045&hgt.customText=http://www.mircode.org/mircode/gencode_mirsites_highconsfamilies.bed) | 7-mer-m8 | ncRNA | no | 89 % | 43 % | 0 % |
| miR-205/205ab | [**chr7:27245098**](http://genome.ucsc.edu/cgi-bin/hgTracks?db=hg19&wgEncodeGencodeSuper=show&wgEncodeGencodeV12ViewGenes=full&wgEncodeGencodeBasicV12_sel=0&wgEncodeGencodeCompV12_sel=1&wgEncodeGencodePseudoGeneV12_sel=1&position=chr7:27245049-27245148&hgt.customText=http://www.mircode.org/mircode/gencode_mirsites_highconsfamilies.bed) | 7-mer-A1 | ncRNA | no | 78 % | 52 % | 0 % |
| miR-214/761/3619-5p | [**chr7:27241061**](http://genome.ucsc.edu/cgi-bin/hgTracks?db=hg19&wgEncodeGencodeSuper=show&wgEncodeGencodeV12ViewGenes=full&wgEncodeGencodeBasicV12_sel=0&wgEncodeGencodeCompV12_sel=1&wgEncodeGencodePseudoGeneV12_sel=1&position=chr7:27241012-27241111&hgt.customText=http://www.mircode.org/mircode/gencode_mirsites_highconsfamilies.bed) | 7-mer-m8 | ncRNA | no | 67 % | 9 % | 0 % |
| miR-216a | [**chr7:27242376**](http://genome.ucsc.edu/cgi-bin/hgTracks?db=hg19&wgEncodeGencodeSuper=show&wgEncodeGencodeV12ViewGenes=full&wgEncodeGencodeBasicV12_sel=0&wgEncodeGencodeCompV12_sel=1&wgEncodeGencodePseudoGeneV12_sel=1&position=chr7:27242327-27242426&hgt.customText=http://www.mircode.org/mircode/gencode_mirsites_highconsfamilies.bed) | 7-mer-m8 | ncRNA | no | 67 % | 13 % | 0 % |
| miR-218/218a | [**chr7:27246682**](http://genome.ucsc.edu/cgi-bin/hgTracks?db=hg19&wgEncodeGencodeSuper=show&wgEncodeGencodeV12ViewGenes=full&wgEncodeGencodeBasicV12_sel=0&wgEncodeGencodeCompV12_sel=1&wgEncodeGencodePseudoGeneV12_sel=1&position=chr7:27246633-27246732&hgt.customText=http://www.mircode.org/mircode/gencode_mirsites_highconsfamilies.bed) | 7-mer-A1 | ncRNA | no | 56 % | 0 % | 0 % |
| miR-223 | [**chr7:27240529**](http://genome.ucsc.edu/cgi-bin/hgTracks?db=hg19&wgEncodeGencodeSuper=show&wgEncodeGencodeV12ViewGenes=full&wgEncodeGencodeBasicV12_sel=0&wgEncodeGencodeCompV12_sel=1&wgEncodeGencodePseudoGeneV12_sel=1&position=chr7:27240480-27240579&hgt.customText=http://www.mircode.org/mircode/gencode_mirsites_highconsfamilies.bed) | 7-mer-A1 | ncRNA | no | 56 % | 0 % | 0 % |
| miR-26ab/1297/4465 | [**chr7:27241702**](http://genome.ucsc.edu/cgi-bin/hgTracks?db=hg19&wgEncodeGencodeSuper=show&wgEncodeGencodeV12ViewGenes=full&wgEncodeGencodeBasicV12_sel=0&wgEncodeGencodeCompV12_sel=1&wgEncodeGencodePseudoGeneV12_sel=1&position=chr7:27241653-27241752&hgt.customText=http://www.mircode.org/mircode/gencode_mirsites_highconsfamilies.bed) | 7-mer-A1 | ncRNA | no | 78 % | 0 % | 0 % |
| miR-27abc/27a-3p | [**chr7:27241103**](http://genome.ucsc.edu/cgi-bin/hgTracks?db=hg19&wgEncodeGencodeSuper=show&wgEncodeGencodeV12ViewGenes=full&wgEncodeGencodeBasicV12_sel=0&wgEncodeGencodeCompV12_sel=1&wgEncodeGencodePseudoGeneV12_sel=1&position=chr7:27241054-27241153&hgt.customText=http://www.mircode.org/mircode/gencode_mirsites_highconsfamilies.bed) | 7-mer-m8 | ncRNA | no | 56 % | 4 % | 0 % |
| miR-29abcd | [**chr7:27240994**](http://genome.ucsc.edu/cgi-bin/hgTracks?db=hg19&wgEncodeGencodeSuper=show&wgEncodeGencodeV12ViewGenes=full&wgEncodeGencodeBasicV12_sel=0&wgEncodeGencodeCompV12_sel=1&wgEncodeGencodePseudoGeneV12_sel=1&position=chr7:27240945-27241044&hgt.customText=http://www.mircode.org/mircode/gencode_mirsites_highconsfamilies.bed) | 7-mer-m8 | ncRNA | no | 56 % | 0 % | 0 % |
| miR-29abcd | [**chr7:27242281**](http://genome.ucsc.edu/cgi-bin/hgTracks?db=hg19&wgEncodeGencodeSuper=show&wgEncodeGencodeV12ViewGenes=full&wgEncodeGencodeBasicV12_sel=0&wgEncodeGencodeCompV12_sel=1&wgEncodeGencodePseudoGeneV12_sel=1&position=chr7:27242232-27242331&hgt.customText=http://www.mircode.org/mircode/gencode_mirsites_highconsfamilies.bed) | 7-mer-m8 | ncRNA | no | 44 % | 0 % | 0 % |
| miR-29abcd | [**chr7:27246053**](http://genome.ucsc.edu/cgi-bin/hgTracks?db=hg19&wgEncodeGencodeSuper=show&wgEncodeGencodeV12ViewGenes=full&wgEncodeGencodeBasicV12_sel=0&wgEncodeGencodeCompV12_sel=1&wgEncodeGencodePseudoGeneV12_sel=1&position=chr7:27246004-27246103&hgt.customText=http://www.mircode.org/mircode/gencode_mirsites_highconsfamilies.bed) | 7-mer-A1 | ncRNA | no | 78 % | 39 % | 0 % |
| miR-30abcdef/30abe-5p/384-5p | [**chr7:27241939**](http://genome.ucsc.edu/cgi-bin/hgTracks?db=hg19&wgEncodeGencodeSuper=show&wgEncodeGencodeV12ViewGenes=full&wgEncodeGencodeBasicV12_sel=0&wgEncodeGencodeCompV12_sel=1&wgEncodeGencodePseudoGeneV12_sel=1&position=chr7:27241890-27241989&hgt.customText=http://www.mircode.org/mircode/gencode_mirsites_highconsfamilies.bed) | 7-mer-m8 | ncRNA | no | 67 % | 78 % | 0 % |
| miR-31 | [**chr7:27246468**](http://genome.ucsc.edu/cgi-bin/hgTracks?db=hg19&wgEncodeGencodeSuper=show&wgEncodeGencodeV12ViewGenes=full&wgEncodeGencodeBasicV12_sel=0&wgEncodeGencodeCompV12_sel=1&wgEncodeGencodePseudoGeneV12_sel=1&position=chr7:27246419-27246518&hgt.customText=http://www.mircode.org/mircode/gencode_mirsites_highconsfamilies.bed) | 7-mer-m8 | ncRNA | no | 56 % | 22 % | 0 % |
| miR-103a/107/107ab | [**chr7:27246574**](http://genome.ucsc.edu/cgi-bin/hgTracks?db=hg19&wgEncodeGencodeSuper=show&wgEncodeGencodeV12ViewGenes=full&wgEncodeGencodeBasicV12_sel=0&wgEncodeGencodeCompV12_sel=1&wgEncodeGencodePseudoGeneV12_sel=1&position=chr7:27246525-27246624&hgt.customText=http://www.mircode.org/mircode/gencode_mirsites_highconsfamilies.bed) | 7-mer-A1 | ncRNA | no | 22 % | 30 % | 0 % |
| miR-124/124ab/506 | [**chr7:27241913**](http://genome.ucsc.edu/cgi-bin/hgTracks?db=hg19&wgEncodeGencodeSuper=show&wgEncodeGencodeV12ViewGenes=full&wgEncodeGencodeBasicV12_sel=0&wgEncodeGencodeCompV12_sel=1&wgEncodeGencodePseudoGeneV12_sel=1&position=chr7:27241864-27241963&hgt.customText=http://www.mircode.org/mircode/gencode_mirsites_highconsfamilies.bed) | 7-mer-m8 | ncRNA | no | 56 % | 0 % | 0 % |
| miR-124/124ab/506 | [**chr7:27245396**](http://genome.ucsc.edu/cgi-bin/hgTracks?db=hg19&wgEncodeGencodeSuper=show&wgEncodeGencodeV12ViewGenes=full&wgEncodeGencodeBasicV12_sel=0&wgEncodeGencodeCompV12_sel=1&wgEncodeGencodePseudoGeneV12_sel=1&position=chr7:27245347-27245446&hgt.customText=http://www.mircode.org/mircode/gencode_mirsites_highconsfamilies.bed) | 7-mer-m8 | ncRNA | no | 56 % | 26 % | 0 % |
| miR-338/338-3p | [**chr7:27241185**](http://genome.ucsc.edu/cgi-bin/hgTracks?db=hg19&wgEncodeGencodeSuper=show&wgEncodeGencodeV12ViewGenes=full&wgEncodeGencodeBasicV12_sel=0&wgEncodeGencodeCompV12_sel=1&wgEncodeGencodePseudoGeneV12_sel=1&position=chr7:27241136-27241235&hgt.customText=http://www.mircode.org/mircode/gencode_mirsites_highconsfamilies.bed) | 7-mer-A1 | ncRNA | no | 67 % | 9 % | 0 % |
| miR-338/338-3p | [**chr7:27244821**](http://genome.ucsc.edu/cgi-bin/hgTracks?db=hg19&wgEncodeGencodeSuper=show&wgEncodeGencodeV12ViewGenes=full&wgEncodeGencodeBasicV12_sel=0&wgEncodeGencodeCompV12_sel=1&wgEncodeGencodePseudoGeneV12_sel=1&position=chr7:27244772-27244871&hgt.customText=http://www.mircode.org/mircode/gencode_mirsites_highconsfamilies.bed) | 7-mer-A1 | ncRNA | no | 56 % | 9 % | 0 % |
| miR-33ab/33-5p | [**chr7:27240959**](http://genome.ucsc.edu/cgi-bin/hgTracks?db=hg19&wgEncodeGencodeSuper=show&wgEncodeGencodeV12ViewGenes=full&wgEncodeGencodeBasicV12_sel=0&wgEncodeGencodeCompV12_sel=1&wgEncodeGencodePseudoGeneV12_sel=1&position=chr7:27240910-27241009&hgt.customText=http://www.mircode.org/mircode/gencode_mirsites_highconsfamilies.bed) | 7-mer-A1 | ncRNA | no | 67 % | 0 % | 0 % |
| miR-34ac/34bc-5p/449abc/449c-5p | [**chr7:27241784**](http://genome.ucsc.edu/cgi-bin/hgTracks?db=hg19&wgEncodeGencodeSuper=show&wgEncodeGencodeV12ViewGenes=full&wgEncodeGencodeBasicV12_sel=0&wgEncodeGencodeCompV12_sel=1&wgEncodeGencodePseudoGeneV12_sel=1&position=chr7:27241735-27241834&hgt.customText=http://www.mircode.org/mircode/gencode_mirsites_highconsfamilies.bed) | 7-mer-A1 | ncRNA | no | 56 % | 39 % | 0 % |
| miR-125a-5p/125b-5p/351/670/4319 | [**chr7:27245798**](http://genome.ucsc.edu/cgi-bin/hgTracks?db=hg19&wgEncodeGencodeSuper=show&wgEncodeGencodeV12ViewGenes=full&wgEncodeGencodeBasicV12_sel=0&wgEncodeGencodeCompV12_sel=1&wgEncodeGencodePseudoGeneV12_sel=1&position=chr7:27245749-27245848&hgt.customText=http://www.mircode.org/mircode/gencode_mirsites_highconsfamilies.bed) | 8-mer | ncRNA | no | 44 % | 43 % | 0 % |
| miR-455-5p | [**chr7:27241156**](http://genome.ucsc.edu/cgi-bin/hgTracks?db=hg19&wgEncodeGencodeSuper=show&wgEncodeGencodeV12ViewGenes=full&wgEncodeGencodeBasicV12_sel=0&wgEncodeGencodeCompV12_sel=1&wgEncodeGencodePseudoGeneV12_sel=1&position=chr7:27241107-27241206&hgt.customText=http://www.mircode.org/mircode/gencode_mirsites_highconsfamilies.bed) | 7-mer-m8 | ncRNA | no | 67 % | 4 % | 0 % |
| miR-128/128ab | [**chr7:27241102**](http://genome.ucsc.edu/cgi-bin/hgTracks?db=hg19&wgEncodeGencodeSuper=show&wgEncodeGencodeV12ViewGenes=full&wgEncodeGencodeBasicV12_sel=0&wgEncodeGencodeCompV12_sel=1&wgEncodeGencodePseudoGeneV12_sel=1&position=chr7:27241053-27241152&hgt.customText=http://www.mircode.org/mircode/gencode_mirsites_highconsfamilies.bed) | 7-mer-A1 | ncRNA | no | 56 % | 4 % | 0 % |
| miR-129-5p/129ab-5p | [**chr7:27246439**](http://genome.ucsc.edu/cgi-bin/hgTracks?db=hg19&wgEncodeGencodeSuper=show&wgEncodeGencodeV12ViewGenes=full&wgEncodeGencodeBasicV12_sel=0&wgEncodeGencodeCompV12_sel=1&wgEncodeGencodePseudoGeneV12_sel=1&position=chr7:27246390-27246489&hgt.customText=http://www.mircode.org/mircode/gencode_mirsites_highconsfamilies.bed) | 7-mer-A1 | ncRNA | no | 33 % | 35 % | 0 % |
| miR-129-5p/129ab-5p | [**chr7:27246600**](http://genome.ucsc.edu/cgi-bin/hgTracks?db=hg19&wgEncodeGencodeSuper=show&wgEncodeGencodeV12ViewGenes=full&wgEncodeGencodeBasicV12_sel=0&wgEncodeGencodeCompV12_sel=1&wgEncodeGencodePseudoGeneV12_sel=1&position=chr7:27246551-27246650&hgt.customText=http://www.mircode.org/mircode/gencode_mirsites_highconsfamilies.bed) | 7-mer-A1 | ncRNA | no | 78 % | 0 % | 0 % |
| miR-490-3p | [**chr7:27244485**](http://genome.ucsc.edu/cgi-bin/hgTracks?db=hg19&wgEncodeGencodeSuper=show&wgEncodeGencodeV12ViewGenes=full&wgEncodeGencodeBasicV12_sel=0&wgEncodeGencodeCompV12_sel=1&wgEncodeGencodePseudoGeneV12_sel=1&position=chr7:27244436-27244535&hgt.customText=http://www.mircode.org/mircode/gencode_mirsites_highconsfamilies.bed) | 7-mer-m8 | ncRNA | no | 56 % | 0 % | 0 % |
| miR-490-3p | [**chr7:27245662**](http://genome.ucsc.edu/cgi-bin/hgTracks?db=hg19&wgEncodeGencodeSuper=show&wgEncodeGencodeV12ViewGenes=full&wgEncodeGencodeBasicV12_sel=0&wgEncodeGencodeCompV12_sel=1&wgEncodeGencodePseudoGeneV12_sel=1&position=chr7:27245613-27245712&hgt.customText=http://www.mircode.org/mircode/gencode_mirsites_highconsfamilies.bed) | 7-mer-m8 | ncRNA | no | 11 % | 0 % | 0 % |
