# Supplementary material for: fMiRNA-192 and miRNA-204 Directly Suppress lncRNA HOTTIP and Interrupt GLS1-Mediated Glutaminolysis in Hepatocellular Carcinoma
Source: PLoS Genet. 2015 Dec 28;11(12):e1005726. doi: 10.1371/journal.pgen.1005726 (PMC4692503; doi:10.1371/journal.pgen.1005726)

**Supplementary Table 2**. Gene Ontolog analyses with 19 differentially expressed genes

| **geneset name** | **# genes in geneset (K)** | **# genes in overlap (k)** | **k/K** | **p value** | **Benjamini q-value** |
| --- | --- | --- | --- | --- | --- |
| regulation of cell proliferation | 787 | 4 | 0.005 | 0.0362 | 1 |
| regulation of apoptosis | 804 | 4 | 0.005 | 0.0382 | 0.998 |
| regulation of programmed cell death | 812 | 4 | 0.005 | 0.0392 | 0.984 |
| regulation of cell death | 815 | 4 | 0.005 | 0.0396 | 0.956 |
| negative regulation of cell proliferation | 361 | 3 | 0.008 | 0.0456 | 0.945 |

**Supplementary Table 3.** The relevant clinic-pathological characteristics of HCC Cases (*n*=48)

| **Characteristics** | **No.** | **%** |
| --- | --- | --- |
| Age (years)  ≤57  >57  Sex  Female  Male  HBsAg  Negative  Positive  Cirrhosis  No  Yes  ALT (U/L)  ≤75  >75  AFP (ng/mL)  ≤20  >20  Tumor size (cm)  ≤5  >5  Tumor number  Single  Multiple  TNM stage  I/II  III/IV | 24  24  6  42  5  43  7  41  39  9  15  33  22  26  39  9  37  11 | 50.0  50.0  12.5  87.5  10.4  89.6  14.6  85.4  81.3  18.7  31.3  68.7  45.8  54.2  81.3  18.7  77.1  22.9 |

HBsAg, hepatitis B surface antigen; AFP, alpha-fetoprotein; ALT, alanine aminotransferase; TNM, tumor-node-metastasis


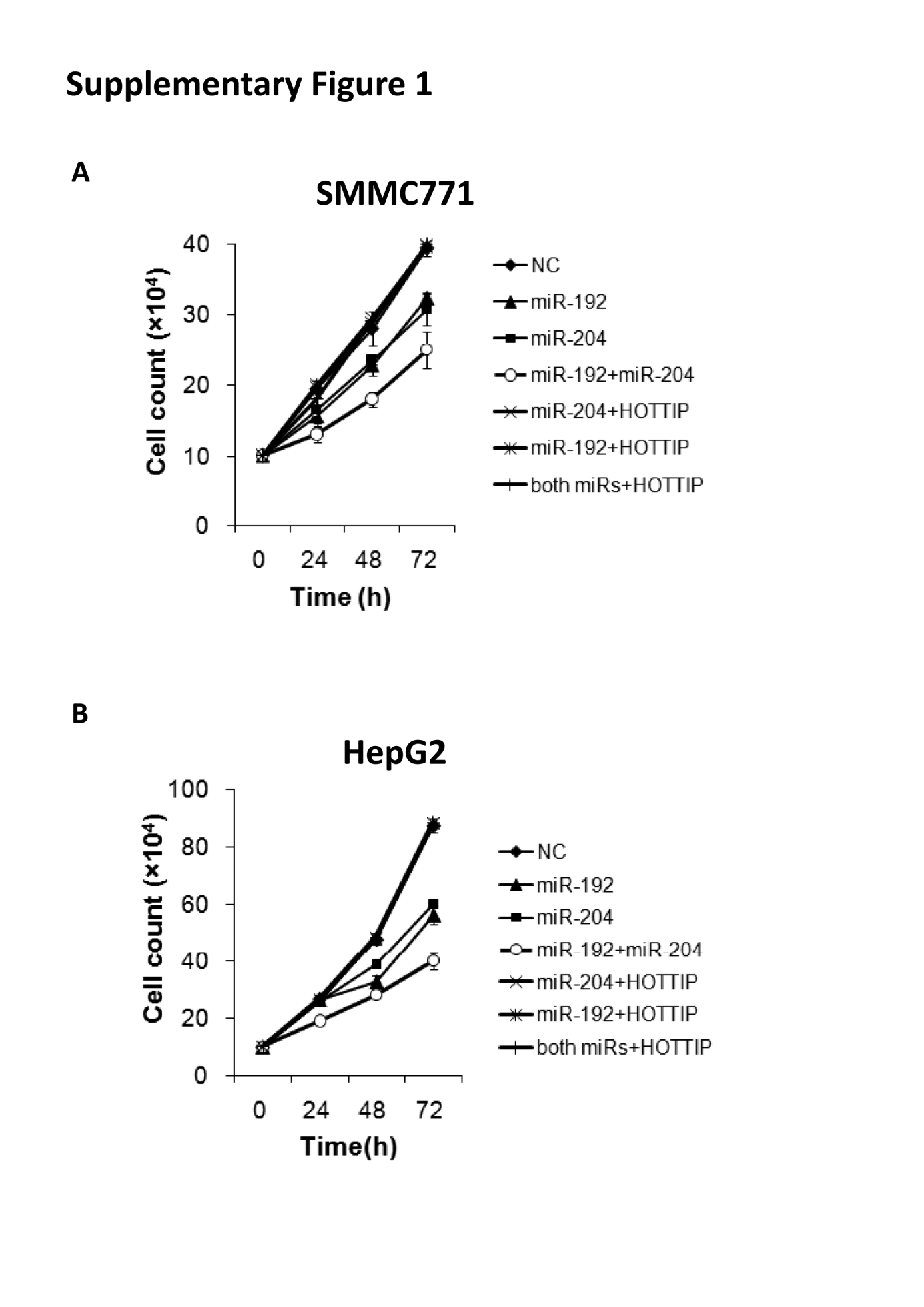

Supplement: S2 Table — (DOCX) [file pgen.1005726.s002.docx]
