# Supplementary figures and images for: fMiRNA-192 and miRNA-204 Directly Suppress lncRNA HOTTIP and Interrupt GLS1-Mediated Glutaminolysis in Hepatocellular Carcinoma
Source: PLoS Genet. 2015 Dec 28;11(12):e1005726. doi: 10.1371/journal.pgen.1005726 (PMC4692503; doi:10.1371/journal.pgen.1005726)

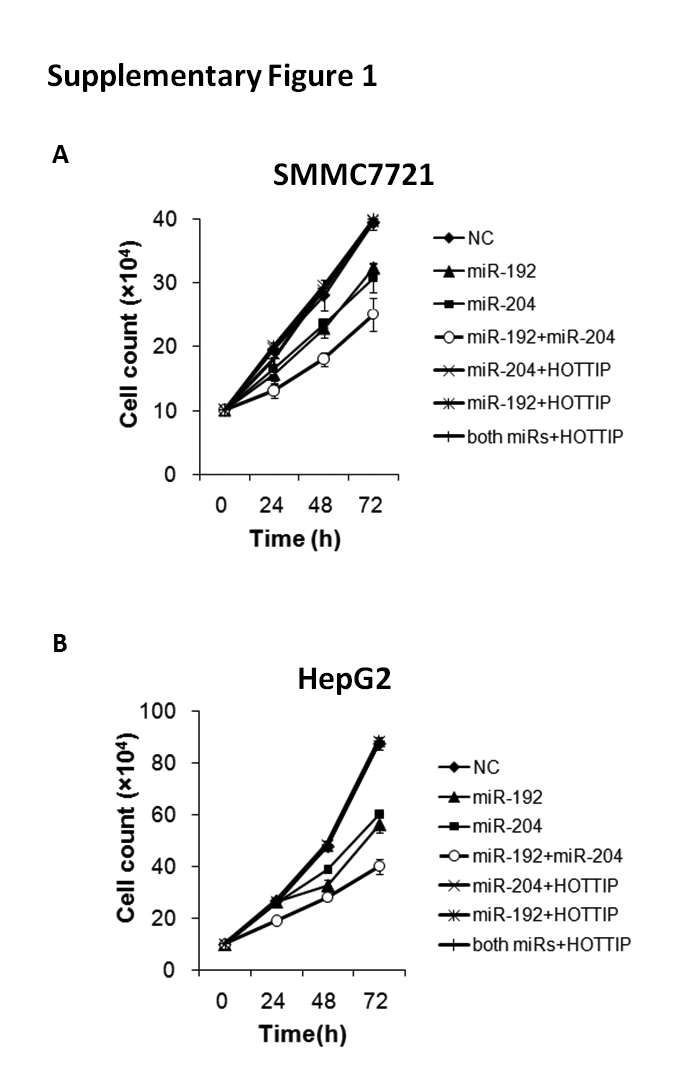

Supplement: S1 Fig — A. Rescue assays in SMMC7721 cells. B. Rescue assays in HepG2 cells. (TIF) [file pgen.1005726.s004.tif]

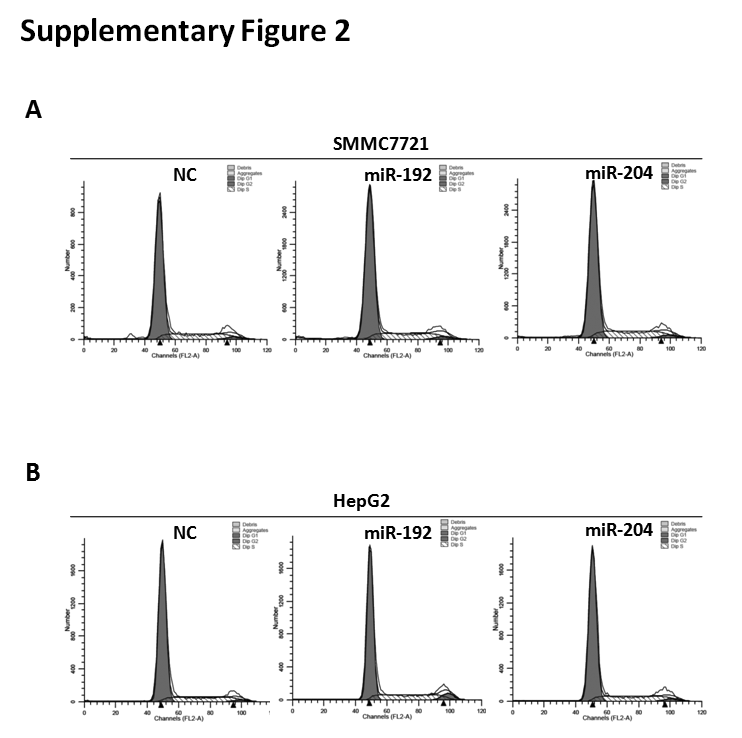

Supplement: S2 Fig — A. Cell cycle analyses in SMMC7721 cells. B. Cell cycle analyses in HepG2 cells. (TIFF) [file pgen.1005726.s005.tiff]

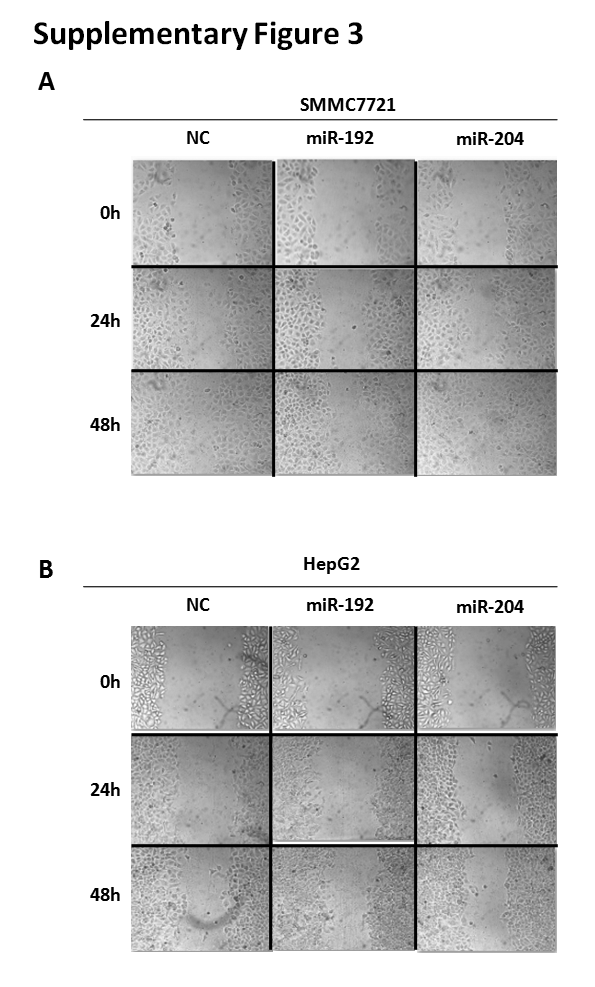

Supplement: S3 Fig — A. Wound healing assays in SMMC7721 cells. B. Wound healing assays in HepG2 cells. (TIFF) [file pgen.1005726.s006.tiff]

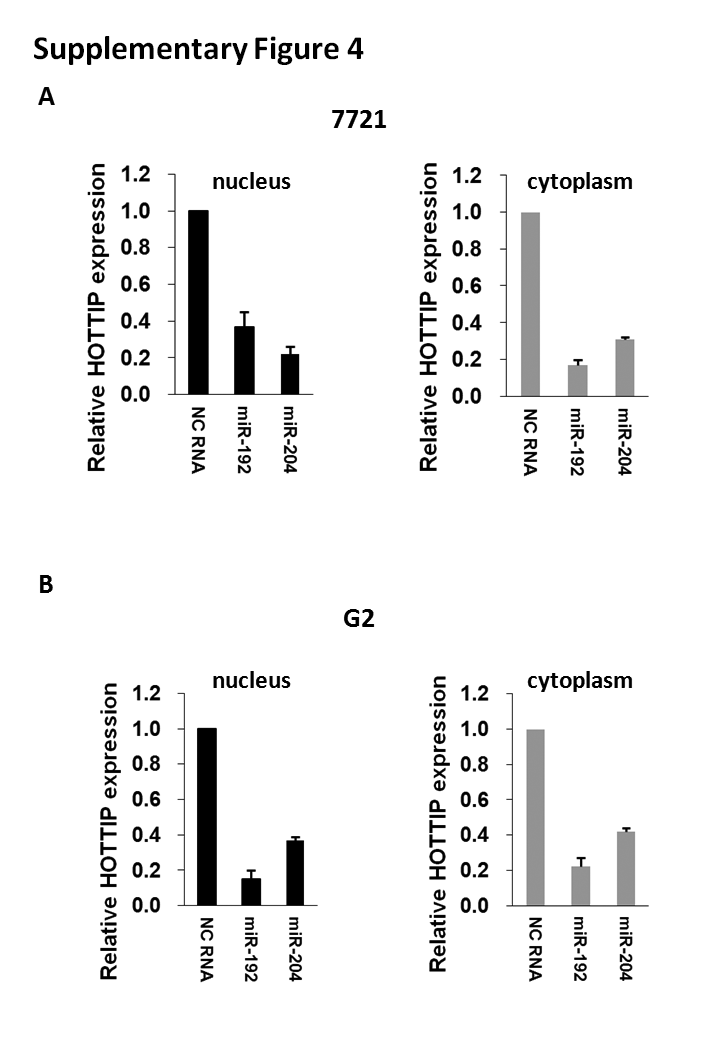

Supplement: S4 Fig — (TIFF) [file pgen.1005726.s007.tiff]

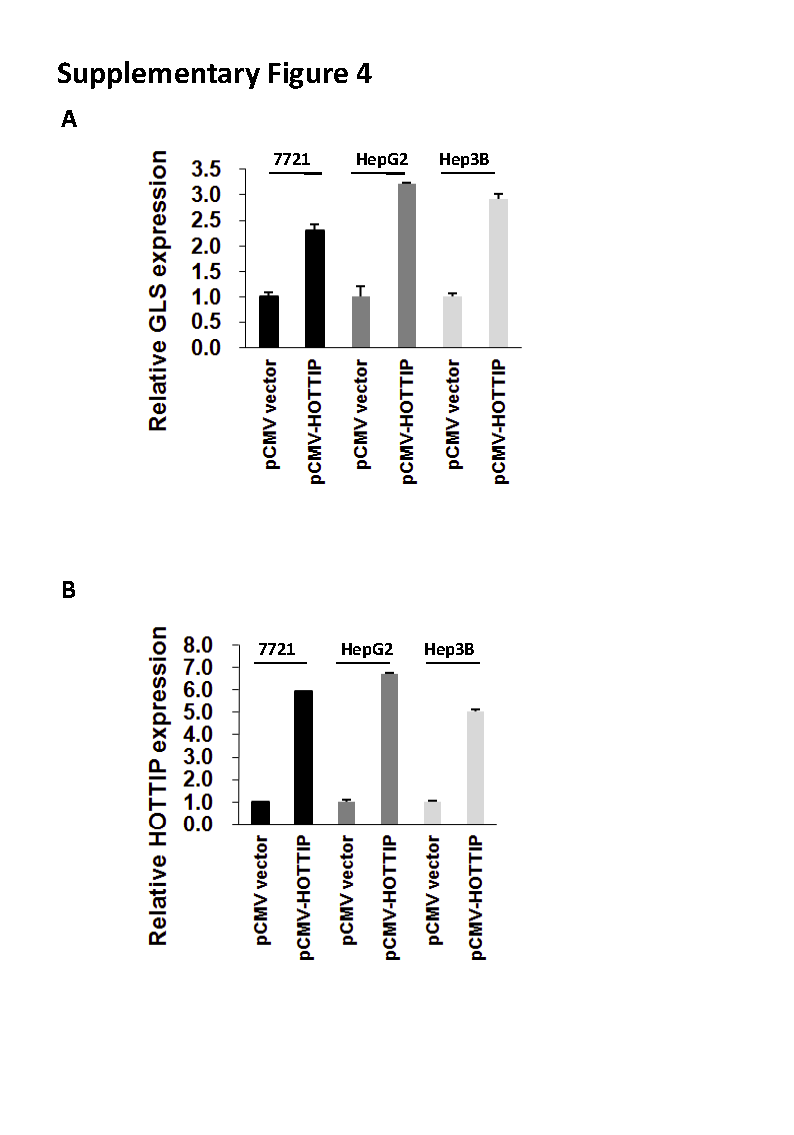

Supplement: S5 Fig — A. GLS1 expression in HCC cells. B. HOTTIP expression in HCC cells. (TIFF) [file pgen.1005726.s008.tiff]
